# Interhemispheric Cortico-Cortical Pathway for Sequential Bimanual Movements in Mice

**DOI:** 10.1523/ENEURO.0200-21.2021

**Published:** 2021-08-24

**Authors:** Minju Jeong, Hyeonsu Lee, Youngsoo Kim, Eric Hou-Jen Wang, Se-Bum Paik, Byung Kook Lim, Daesoo Kim

**Affiliations:** 1 Department of Biological Sciences, Korea Advanced Institute of Science and Technology, Daejeon 34141, Republic of Korea; 2 Department of Bio and Brain Engineering, Korea Advanced Institute of Science and Technology, Daejeon 34141, Republic of Korea; 3 Program of Brain and Cognitive Engineering, Korea Advanced Institute of Science and Technology, Daejeon 34141, Republic of Korea; 4 Neurobiology Section, Division of Biological Sciences, University of California, San Diego, La Jolla, CA 92093; 5 Biomedical Sciences Graduate Program, University of California, San Diego, La Jolla, CA 92093

**Keywords:** cortico-cortical pathway, interhemispheric projection, sequential bimanual movements

## Abstract

Animals precisely coordinate their left and right limbs for various adaptive purposes. While the left and right limbs are clearly controlled by different cortical hemispheres, the neural mechanisms that determine the action sequence between them remains elusive. Here, we have established a novel head-fixed bimanual-press (biPress) sequence task in which mice sequentially press left and right pedals with their forelimbs in a predetermined order. Using this motor task, we found that the motor cortical neurons responsible for the first press (1P) also generate independent motor signals for the second press (2P) by the opposite forelimb during the movement transitions between forelimbs. Projection-specific calcium imaging and optogenetic manipulation revealed these motor signals are transferred from one motor cortical hemisphere to the other via corticocortical projections. Together, our results suggest the motor cortices coordinate sequential bimanual movements through corticocortical pathways.

## Significance Statement

The orchestration of left and right limbs is required to perform complex behaviors in our daily life. It has been presumed that the two cortical hemispheres might interact with each other through the callosal pathways. Yet, the exact neural circuit mechanisms how information exchange of limb movements occurs between the two hemispheres still remain unclear. Here, we establish a novel motor task in head-fixed mice to perform sequentially press left and right pedals, respectively. We then found out that the transfer of motor signals via corticocortical pathways arisen during the movement transition period is essential for precise coordination of left and right limbs. Our findings implicate the functional roles of corticocortical pathways in complex motor behaviors.

## Introduction

The intricate coordination between our limbs underlies much of our daily tasks, performing musical instruments, playing sports, or manipulating various objects. Specifically, the left and right limbs need to be elaborately coordinated, with smooth transition between action sequences and timing (Lashley, 1951; Diedrichsen and Kornysheva, 2015), to perform complex behaviors. Although it is well established that each hemisphere of the mammalian brain can control the opposite limb movements (Penfield and Rasmussen, 1950; Tanji et al., 1988; Lemon, 2008), it remains unclear how these hemispheres interact to coordinate temporally precise movements that require both left and right limbs.

Previous observations have reported that patients with a damaged callosal pathway showing symptoms of aberrant spatiotemporal coordination between left and right hands (Eliassen et al., 1999; Kennerley et al., 2002; Bonzano et al., 2008), suggesting that the callosal pathway seems to be important for bimanual coordination (Bloom and Hynd, 2005; Gooijers and Swinnen, 2014). As the callosum is a major pathway for interhemispheric corticocortical connectivity (Wahl et al., 2007; Zhou et al., 2013; Oh et al., 2014; Zingg et al., 2014), the prevailing assumption, yet uncharacterized, is that interhemispheric corticocortical pathways regulate these bimanual movements.

Although recent developments in neuro-imaging, optogenetics, and behavioral measurements have advanced our understanding of the neural substrate of unilateral movements in rodents (Makino et al., 2017; Terada et al., 2018; Mayrhofer et al., 2019), we still understand little about the regulation of sequential bimanual movements. This is, in part, because of a lack of sophisticated behavioral measurements that can reliably constrain sequential bimanual movements. Addressing this problem required us to develop a novel head-fixed, behavioral paradigm that trains mice to sequentially produce, temporally precise bimanual movements. By combining this approach with calcium imaging, *in vivo* extracellular recording, projection-specific viral targeting, and optogenetics, we found that sequential transfer of motor signals through interhemispheric corticocortical projections contribute to the sequential bimanual movements.

## Materials and Methods

### Animals

All animal care and experimental procedures were performed in accordance with protocols approved by the directives of the Animal Care and Use Committee of Korea Advanced Institute of Science and Technology (approval number KA2012-04) and the Institutional Animal Care and Use Committee (IACUC) of the University of California, San Diego. Either of male or female C57BL/6J, VGAT-ChR2 (JAX 014548), and Thy1-GCaMP6s (JAX 024275) mice were used. All mice applied for behavioral tests were single-housed under a 12/12 h light/dark cycle with free access to rodent chow, with water restriction initiated after the head-implant surgery.

### Surgery

Mice were anesthetized with 2,2,2-tribromoethanol (20 mg/ml, i.p.; Sigma T48402) or 1–2% isoflurane and placed on a stereotaxic apparatus (Kopf Instruments). For wide-field imaging (WFI) experiments, the skulls of Thy1::GCaMP6s mice were thinned to ∼250–500 μm thick using a dental drill, to obtain high quality images of the cortical surface. Thereafter, a custom-designed head plate was implanted with dental cement (C&B Metabond; Sun Medical Co or Parkell). For *in vivo* single-unit recording experiments, a head plate was implanted to the skull of each C57BL/6J mouse. After the cement hardened, the opened skull was covered with Kwik-Sil (WPI) for protection. For GCaMP photometry experiments, 0.5 μl of premixed AAV9-CamKII0.4-Cre-SV40 (3.87 × 10^13^ particles/ml; University of Pennsylvania) and AAV9-CAG-Flex-GCaMP6f-WPRE-SV40 (2.61 × 10^13^ particles/ml; University of Pennsylvania; 1:1 ratio) viruses were injected into the caudal forelimb area (CFA; AP +0.85; ML –1.1; DV –1.2 mm) of the C57BL/6J mice. After the viral injection, an optic cannula (F1.5-MM-FP-OPTH; Becker & Hickl GmbH) was implanted into the opposite-hemisphere CFA (AP +0.85; ML +1.1; DV –1.0 mm) and a head plate was implanted to the skull. For optogenetic experiments, an optic cannula (MFC_200/230–0.48_1.3 mm_MF1.25_FLT; Doric Lenses Inc.) was inserted into the CFA and a head plate was implanted onto the skull of VGAT::ChR2 and littermate control mice. For optogenetic inhibition of corticocortical afferents, 0.5 μl of retroAAVDJ-Cre and AAVDJ-EF1a-Flex-ArchT-GFP were injected into each CFA of C57BL/6J mice. These two adeno-associated virus (AAV) vectors were generated as previously described (Lim et al., 2012). After viral injection, the optic cannula was implanted into the CFA of the hemisphere that had been injected with the retroAAVDJ-Cre viruses, and a head plate was implanted onto the skull.

### Bimanual-press (biPress) sequence task training

The experimental apparatus was custom-made with a 3D printer (Marv; Canon, Ultimaker S2; Ultimaker) and the software for manipulating the hardware was custom-designed using the Arduino program (Mega2560 boards; Arduino). Water was delivered by a peristaltic pump. The pedal-press state and time were recorded by a custom-designed Python program. When a mouse pushes the pedal down with enough force, it flips a microswitch under the pedal, an Arduino-controlled step motor rotates to lock the pedals into position (Extended Data Fig. 1-1). After each trial, the step motor rotates back in the opposite direction, resetting the retracted pedals. We designed this apparatus in which a mouse cannot re-press the pedal until it is reset to avoid the confounding from double-taps.

**Figure 1.**
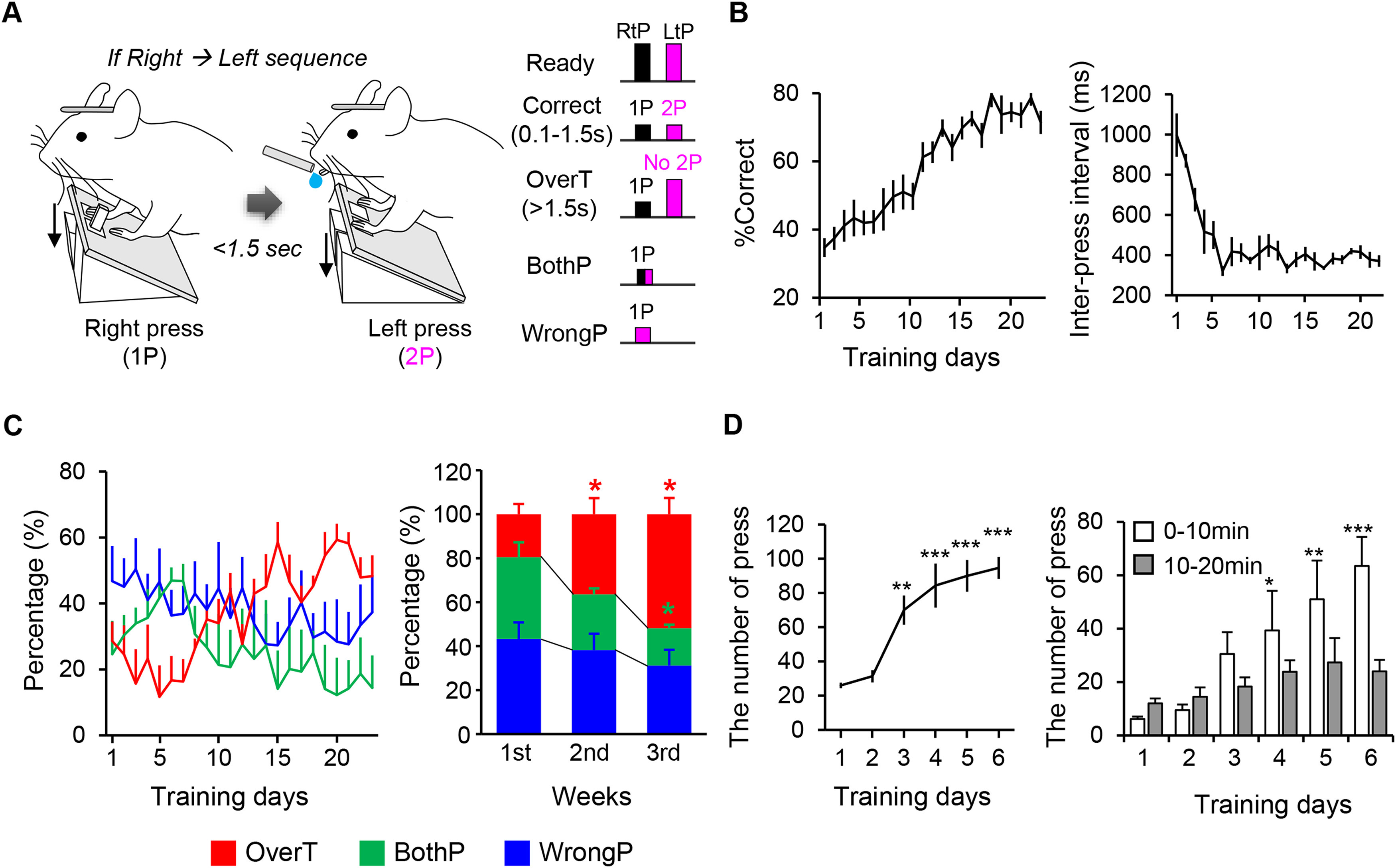
biPress sequence task training. ***A***, Schematic diagram of the biPress motor task. Left, long bars and short bars represent non-pressed and pressed state, respectively. Black and magenta represent 1P and 2P (see also Extended Data Fig. 1-1 for description of pedal movements in each trial). ***B***, left, Behavior performance represented as the percentage of correct trials. Right, The time interval between the 1P and the 2P. ***C***, left, The percentage of each incorrect trial over the total number of incorrect trials during the training period. Right, The fraction of each incorrect trial per week during the training period. ***D***, The number of pedal-press during stage 1 of the biPress task training. Left, The total number of pedal-press in a whole session. Right, The number of pedal-press early (0–10 min) and late (10–20 min) phase of a daily session. All data are represented as mean ± SEM; *p < 0.05, **p <* *0.01, ***p <* *0.001. *Figure Contributions:* Minju Jeong performed the experiments and analyzed the data.

At 3∼5 d after the surgery, mice were water restricted to 1 ml/d for the training, which proceeded via three stages. During stage 1 of the training (<7 d), the mice were water-rewarded for singly pressing the left or right pedal, but not for simultaneously pressing both pedals. The mice were trained only to press the pedals, not to hold them down. Once a mouse exceeded 70 correct trials within 20 min [*n* = 6 mice, one-way RM ANOVA with Tukey’s *post hoc* test, *F*_(5,35)_ *=* 19.127, *p *<* *0.001, *p = *0.002 for day 3, *p *<* *0.001 for days 4–6 compared with day 1 (Fig. 1*D*, left); two-way RM ANOVA, days: *F*_(5,71)_ = 10.974, *p *<* *0.001 (Fig. 1*D*, right)], it advanced to the next stage. In stage 2 of the training (2 d), mice were rewarded for pressing either the left→right or right→left sequence. If a mouse simultaneously pressed both pedals, no water reward was given. Once a mouse exceeded 70 correct trials within 20 min, it advanced to the next stage. We excluded the mice that did not learn the both left and right pedal-press from the next stage. In the final stage of the training (>21 d), the pressing sequence was fixed in one direction. The mice were rewarded for pressing each pedal in the desired sequence within 5–1.5 s. This between-pedal-press interval (1-2P period) was gradually decreased by 0.5 s for every 10 correct trials. If a mouse pressed both pedals at the same time or pressed the wrong side first, the event was regarded as an incorrect trial (bothP and wrongP, respectively). If the mouse pressed the correct-side pedal at first but did not press the other side pedal within the desired interval, it was also regarded as an incorrect trial (overtime fail, overT). A session was conducted for 15 min/d. All trials were voluntarily started without any sensory cue.

10.1523/ENEURO.0200-21.2021.f1-1 Extended Data Figure 1-1 Diagrams showing pedal movements in the correct and incorrect trials. The pedals are retracted after mice press them until the next trial begins. Contra and Ipsi FL stand for contralateral and ipsilateral forelimb, respectively. *Figure Contributions:* Minju Jeong performed the experiments. Download Figure 1-1, TIF file.

### Timed unimanual-press (uniPress) sequence task training

The same experimental apparatus was used as the biPress motor task. Three to 5 d after the surgery, mice were water restricted to 1 ml/d for the training. During the first 5 d, the mice were trained to press the pedal, in which they were water-rewarded for singly pressing either the left or right pedal. Reward-associated pedal was assigned by the location of viral injection, which was either ipsilateral or contralateral to axon terminals of corticocortical projections (Fig. 4*G*). After the single pedal-press, an Arduino-controlled step motor rotates to lock the pedals into position, and it rotates back to reset the pedals after each trial. Once a mouse exceeded 70 correct trials within 20 min, it advanced to be trained to sequentially press the assigned pedal in a fixed-ratio 2 (FR2). In this stage of the training, the step motor rotates to lock the pedals after the mice press the assigned pedal twice. Animals were initially rewarded for pressing the pedal twice between 150 and 1500 ms. Water-reward was not given when the mice press the pedal twice within 150 ms (swift) or only once (over 1500 ms; overT; Fig. 4*G*). We appointed minimum interval to prevent mice from pressing the pedal twice too fast causing insufficient optogenetic effects. A session was conducted for 15 min/d. All trials were voluntarily started without any sensory cue.

### Fluorescent WFI

WFI was performed on day 1or 2 of the training step 3 (beginner) and on day 21 or 22 (expert) using a CMOS camera (FLASH4; C-13 440-20CU; Hamamatsu) system, adapted from a previous study (Kim et al., 2016). The cortical surface was imaged through a 2×/0.08 NA objective (PlanApo N; Olympus). For measurement of GCaMP6 emission, a 470 nm LED (Optogenetics-LED-Blue; Prizmatrix) was connected to the dichroic cube holder using a 1000-μm-diameter 0.50-NA fiber (M59L01; Thorlabs). The light was filtered with a 470 nm bandpass filter (FB470-10; Thorlabs) that was fiber-coupled into the dichroic mirror (FF495-Di03-25 × 36; Semrock). Fluorescence emitted from the cortical surface was passed through a 530 nm bandpass fluorescence emission filter (FF01-520/35-25; Semrock). Images were acquired using HCImage Live (Hamamatsu) at 20 Hz (50 ms/frame) and 192 × 192 pixels. Imaging was started by using the transistor-transistor logic (TTL) signal generated from the Arduino to synchronize the imaging and behavior data.

### *In vivo* single-unit recording

Eectrodes were custom made with four-fold twisted polyimide-coated NiCr wires (PF002005; Kanthal Precision Technology), and each wire was assembled into an electrode interface board (EIB-16 board; Neuralynx) with gold pins (Neuralynx). The electrode-interface boards were assembled into a custom-designed holder. The impedance of each tetrode channel was reduced by electro-coating with colloidal gold using a Nano-Z kit (Neuralynx). The impedance of each recording channel was 300–400 kΩ.

At 2–3 d before the recording, mice were anesthetized with isoflurane (1.5% in oxygen) and head-fixed with the task device. A hole was drilled in the skull above the CFA, filled with artificial CSF (ACSF), covered with a round glass (CS-3R, catalog #64-0720, Warner Instruments), and sealed with Kwik-Sil (World Precision Instruments). On the recording day, the silicon and cover glass were removed, and the electrodes were put on DiI or DiO (Invitrogen) and inserted into the hole. Once a neural signal was successfully detected, the hole was sealed with 1.5% liquid agar to prevent movement from causing the signal to be lost during the task.

All mouse behaviors were recorded using an action-cam (HDR-AS200V; Sony) at 240 fps for detailed analysis of forelimb movements. The 1-2P period for correct trials was set to 1.5 s. Neural signals were recorded with the Cheetah 32-channel acquisition system (Neuralynx) with bandpass filtering at 3–5 kHz. The pedal-press signals obtained from the Arduino board were interconnected with the Cheetah acquisition system as TTL signals for time synchronization.

### Fiber photometry

A single-wavelength time-correlated single photon counting (TCSPC) photometry system (Becker & Hickl GmbH) was used for *in vivo* axon fiber calcium imaging during the biPress motor task. A 488 nm laser (BDL-488-SMC; Becker & Hickl GmbH) was used to excite GCaMP signals at 20 MHz through a single-mode fiber (F100-MM-FC-FET; Becker & Hickl GmbH) connected to a multimode probe (F05-MM-FP-OPTH; Becker & Hickl GmbH). The power measured at the free end of the optic cannula was ∼0.1 mW. Photons emitted from the tissue traversed the multimode fiber before being collected by a photodetector (PMC-100; Becker & Hickl GmbH) controlled by a detector controller (DCC-100; Becker & Hickl GmbH), and the number of photons was counted at 20 Hz. All mouse behaviors were recorded using action-cams at 240 fps. The Arduino board of the task device and the photometry recording system were interconnected for time synchronization. The 1-2P period cutoff for a correct trial was set to 1.5 s.

### Optogenetic behavioral tests

Patch cords (MFP_200/230/900–0.48_1m; Doric Lenses) were connected to the optic cannula and a 589 nm laser (MGL-F-589-100mW; Opto Engine LLC). The laser power was set to ∼10 mW as measured at the tip of the optic cannula using a power sensor (PM100D-S130VC; Thorlab). The light illumination was controlled by the Arduino program. For optogenetic manipulation during the 1-2P period, the laser was turned on right after first press (1P) and turned off right after second press (2P) or at 1.5 s (fixed interval). For optogenetic manipulation during the pre-1P period, the laser was turned on when the retracted pedals were reset and turned off right after the 1P-sided and/or 2P-sided pedals were pressed.

### Data analysis

#### WFI data analysis

Imaging data analysis was performed with customized MATLAB codes (MathWorks). To align brain images from different sessions, a two-dimensional cross-correlation between images was calculated to estimate the amount of spatial shift that should be applied to each image. Images from different mice were aligned using the position of the bregma. To reduce any background noise, each brain image was smoothed with a two-dimensional Gaussian filter (σ = 2 pixels).

The rostral forelimb area (RFA) and CFA regions were determined based on motor maps generated by previous intracortical microstimulation experiments (Tennant et al., 2011; Hira et al., 2013). The coordinates were AP +1.5–2.7; ML ±0.75–1.75 mm for the RFA and AP +0.8 to –0.8; ML ±1.2–2.5 mm for the CFA. The region of visual area (VIS) was determined based on the Allen Brain Atlas and the coordinates were AP –2.59–5.17; ML ±1.23–3.87 mm.

Time series data on the activity in each pixel was filtered between 0.1 and 9.9 Hz. Each correct trial was defined as spanning 1250 ms before 1P to 1250 ms after 2P. For incorrect trials, the trial end was defined as 1250 ms after the time cutoff for an overT trial or 2000 ms after 1P for bothP and wrongP trials. Samples which data lay beyond 3 σ (SD) of the entire dataset were excluded from the analysis.

To obtain ΔF/F values for each pixel, the baseline fluorescence (F) was estimated from the average activity of the non-sample periods. The series average of ΔF/F was smoothed over time with a Gaussian filter (σ = 150 ms). To detect a behavior-related peak, the peak of activity was searched within a −500- to +500-ms window of each behavioral press. Since the calcium signals were weak and showed multiple peaks in the VIS, samples were smoothed with a Gaussian filter (σ = 500 ms) and those showing peak values larger than mean + SD of sample activity were included in the analysis to find a significant peak activity.

#### Behavior video analysis for *in vivo* single-unit and GCaMP photometry

The movement start (MovS) of each event was determined as the time when the animal started to move its forelimb after the rest period. The 1P and 2P events were indicated by red and green LEDs signaled from the Arduino; thus, the interval between the MovS and 1P events could be calculated through frame-by-frame video analysis. We did not specifically classify movement types for the MovS in this study. It included either movements of 1P-related or 2P-related forelimb. A rest period was defined as a period during when the mouse showed no movement for >1 s. Trials without a rest period were excluded from the data analysis.

#### *In vivo* single-unit recording

For single-unit isolation, spikes were extracted and clustered using the Spike Extractor and SpikeSort 3D software packages (Neuralynx). Only high-quality isolated units, which were defined as having Lratio <0.1 and isolation distance >15 (Kim et al., 2017; Lee et al., 2012) were selected for data analysis.

Samples shorter than 5 s were selected for analysis. The instantaneous firing rate was calculated by applying a Gaussian filter (σ = 100 ms) to the observed spike activities. For quantitative comparison of firing patterns, the instantaneous firing rate during the various intervals [i.e., MovS-1P, 1-2P (for a correct trial) or 1P-trial end (for an incorrect trial)] were linearly interpolated to match the activity length by rescaling to 1 s for MovS-1P, to 0.5 s for 1-2P or to 0.6 s for 1P-trial end.

For classification of neural firing patterns, a time window of 100 ms before and after the press timing was considered. The firing rate in each window was z-scored within each neuron. We classified activities for each behavioral event (MovS, 1P, and 2P) into two groups using k-means clustering method because the Silhouette index was maximized for the two groups (*k *=* *2–6). Neurons could be classified into eight groups according to the combination of behavior patterns (Extended Data Fig. 3-1*B*,*C*, *n* = 171).

Principal component analysis of average neural firing rates was performed using MATLAB (MathWorks). To find neurons that encoded correct and incorrect trials differently, the cumulative curves of differences in the firing patterns (ΔFiring Rate, ΔFR) between correct and incorrect trials were classified using k-means clustering into *k* groups (*k *=* *2–6). We then measured the Silhouette index across the clusters to determine the optimal number of clusters, which was two groups. Using the observed neural activities of each group, we have decoded whether each trial was correct or incorrect. A trial performance decoder (determining whether the trial was correct or incorrect) was implemented (fivefold, 200-ms window, 100-ms shift, 50 repetitions) using the support vector machine (SVM) algorithm.

#### Fiber photometry data analysis

All procedures were performed as described for the single-unit data analysis. The total photon count was obtained over 50 ms, and the time series of the fluorescent signals was filtered between 0.1 and 9.9 Hz. To obtain ΔF/F values, the baseline fluorescent signal (F) was estimated from the trial start (Trial-S)–MovS period of each trial and filtered in time with a Gaussian filter (σ = 150 ms).

#### Histology

Mice were deeply anesthetized with 2,2,2-tribromoethanol (20 mg/ml, i.p.; Sigma T48402) or a mixture of ketamine (100 mg/kg) and dexmedetomidine (0.5 mg/kg). The animals were then perfused and fixed with the 0.9% saline and 4% formaldehyde in PBS. Brains were removed, postfixed in 4% formaldehyde overnight at 4°C, sectioned into 50-μm coronal slices using a vibratome (VT1200S; Leica) and mounted onto glass slides with DAPI mounting medium solution (H-1500; Vector Labs). Images were taken under a fluorescent microscope (VS120; Olympus) or a confocal microscope (KAIST Biocore center, LSM780; Zeiss).

#### Statistics

Statistical analyses were performed using SigmaPlot12 (Systat Software) and MATLAB (MathWorks). For WFI analysis, we used Wilcoxon signed-rank test, two-tailed paired *t* test and one-way repeated-measures (RM) ANOVA with Tukey–Kramer multiple comparisons test. For single-unit data analysis, we applied Wilcoxon signed-rank test, Friedman test, two-sample *t* test. For fiber photometry data analysis, we used Wilcoxon signed-rank test. For optogenetic experiments, we used two-tailed paired/unpaired *t* tests, and the nonparametric Wilcoxon signed-rank test and Mann–Whitney *U* test. Normality and equal variance of the data distribution were examined; *p *<* *0.05 was considered statistically significant.

## Results

### Coherent neuronal activities in the two motor cortical hemispheres during the biPress sequence task

We first designed a biPress sequence task in which head-fixed mice were trained only to press the right (1P) and then left (2P) pedals, sequentially, or vice versa (Fig. 1*A*; Extended Data Fig. 1-1; Movies 1, 2, 3, 4). The mice were initially water-rewarded when they completed 2P within 5 s after 1P. The allowed time interval between 1P and 2P (the 1-2P period) was shortened by 0.5 s for every 10 correct trials, down to a minimal duration of 1.5 s (Fig. 1*A*). The forelimb for 1P was positioned to the pedal and the other forelimb was placed into the floor to be balanced. These positions were switched each other for 2P (Movie 1). Incorrect trials included instances of mice not pressing 2P within the desired time interval (overT), pressing both pedals together (bothP), and pressing the wrong side as 1P (wrongP; Fig. 1*A*; Movies 2, 3, 4). Although the mice showed similar movement patterns for 1P but did not show any movements after 1P causing overT incorrect trials (Movie 2). Meanwhile, in bothP trials, the mice positioned both forelimbs to each pedal and pressed them almost simultaneously (Movie 3). In wrongP incorrect trials, the 1P-related forelimb was moved but not placed it into the pedal, and then the other forelimb was performed the pedal-press (Movie 4). After three weeks of training, the mice reduced unnecessary movements and showed a higher ratio of correct trials than incorrect trials and decreases in the length and variability of the 1-2P period compared with those observed during the beginning phases of the training (*n* = 6 mice, left, one-way RM ANOVA with Tukey’s *post hoc* test, *F*_(22,137)_ = 14.953, *p *<* *0.001, right, one-way RM ANOVA with Tukey’s *post hoc* test, χ^2^ = 62.094 with 22 degrees of freedom, *p *<* *0.001; Fig. 1*B*). As the mice learned to sequentially press the pedals during the training, the ratio of overT to total incorrect trials also increased compared with that of the other incorrect trial types (*n* = 6 mice, left, two-way RM ANOVA, *F*_(22,413)_ = 3.839, *p *<* *0.001 for days, *F*_(44,413)_ = 1.905, *p *=* *0.001 for days × type, right, two-tailed paired *t* test, orange, *p *=* *0.0273 for day 1 vs day 3, Wilcoxon signed-rank test, green, *p *=* *0.031 for day 1 vs day 2; two-tailed paired *t* test, green, *p *=* *0.0185 for day 1 vs day 3; Fig. 1*C*), suggesting that the mice successfully learned the biPress motor task.

Movie 1. Example movie of a mouse performing the biPress task. Movie shows representative correct trial in the same mouse with the view of each forelimb. The movie is played 1× speed and then 0.2× slow motion. The mouse was trained to press the pedals in a right→left sequence. The 1P, 2P, and correct trial were indicated by the red, green, and yellow LED, respectively. These LEDs were embedded only during the recording day for analysis. 10.1523/ENEURO.0200-21.2021.video.1

Movie 2. Example movie of a mouse performing the biPress task. Movie shows representative overT trial in the same mouse with the view of each forelimb. The movie is played 1× speed and then 0.2× slow motion. The mouse was trained to press the pedals in a right→left sequence. The 1P, 2P, and correct trial were indicated by the red, green, and yellow LED, respectively. These LEDs were embedded only during the recording day for analysis. 10.1523/ENEURO.0200-21.2021.video.2

Movie 3. Example movie of a mouse performing the biPress task. Movie shows representative bothP trial in the same mouse with the view of each forelimb. The movie is played 1× speed and then 0.2× slow motion. The mouse was trained to press the pedals in a right→left sequence. The 1P, 2P, and correct trial were indicated by the red, green, and yellow LED, respectively. These LEDs were embedded only during the recording day for analysis. 10.1523/ENEURO.0200-21.2021.video.3

Movie 4. Example movie of a mouse performing the biPress task. Movie shows representative wrongP trial in the same mouse with the view of each forelimb. The movie is played 1× speed and then 0.2× slow motion. The mouse was trained to press the pedals in a right→left sequence. The 1P, 2P, and correct trial were indicated by the red, green, and yellow LED, respectively. These LEDs were embedded only during the recording day for analysis. 10.1523/ENEURO.0200-21.2021.video.4

**Figure 2.**
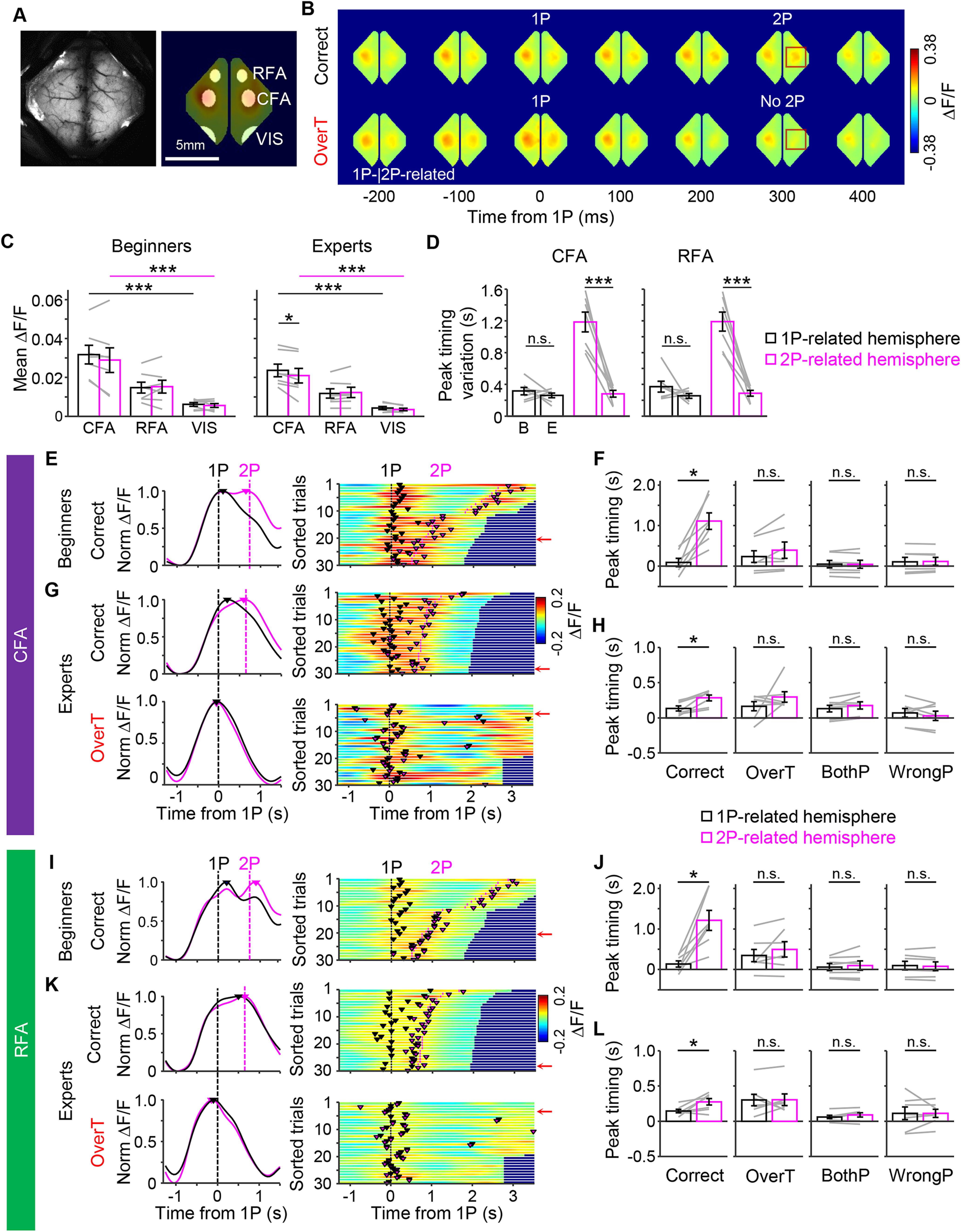
Sequential Ca^2+^ activities between the two motor cortical areas during the biPress sequence task in head-fixed mice. ***A***, left, A field of view of wide-field Ca^2+^ imaging in the mouse cortex. Right, Motor area (CFA, RFA) and VIS were examined. ***B***, Sample patterns of cortical activity represented as changes in GCaMP6 signals during the biPress task. Red squares indicate differences between correct and incorrect trials. ***C***, The average ΔF/F in the CFA, RFA, and VIS. ***D***, Peak timing variation across the brain regions. Left, Beginners versus experts in the CFA. Right, Beginners versus experts in the RFA. B, beginners; E, experts. ***E***, ***G***, Representative activity patterns in the CFA of beginner (***E***) and expert (***G***) mice. Left, Sample activity pattern in a single trial. Black and magenta curves represent normalized ΔF/F activities of the 1P-related and 2P-related hemispheres, respectively. Vertical dashed lines indicate the time points of 1P (black) and 2P (magenta). Colored triangles represent the activity peak timing. Right, Population activities shown as a heatmap. Colored triangles and vertical lines represent the activity peak timing and the timing of 1P (black) and 2P (magenta), respectively. Trials were sorted by the duration. Red arrows indicate the sample trials presented in the left panel. ***F***, ***H***, Comparison between the peak timings of 1P-CFA and 2P-CFA of the beginner (***F***) and expert (***H***) mice. Black and magenta boxes represent the averaged activity peak timings in the 1P-related and 2P-related hemispheres, respectively. Gray lines represent individual data. ***I***, ***K***, Representative activity patterns in the RFA of beginner (***I***) and expert (***K***) mice. ***J***, ***L***, Comparison between the peak timings of 1P-RFA and 2P-RFA of the beginner (***J***) and expert (***L***) mice. Same format as ***E–H***. All error bars represent SEM; *p < 0.05, ***p < 0.001, n.s. not significant Comparisons of ΔF/F between the 1P-related and 2P-related hemispheres were shown in Extended Data Figure 2-1*A*,*B*. Comparisons of peak timings in the VIS were shown in Extended Data Figure 2-1*C*. *Figure Contributions:* Minju Jeong performed the experiments. Hyeonsu Lee analyzed the data.

Next, to investigate the cortical dynamics of the hemispheres, we used Thy1-GCaMP6s mice harboring the genetically encoded Ca^2+^ indicator, GCaMP6s, in their cortical neurons (Wekselblatt et al., 2016; Makino et al., 2017) and performed wide-field Ca^2+^ imaging (WFI) on the left and right cortices during the biPress task training (Fig. 2). The 1P-related and 2P-related Ca^2+^ signals increased mainly in the cortical areas known as the RFA and the CFA (Tennant et al., 2011; Hira et al., 2013) during the task (Fig. 2*A–C*). Moreover, the CFA showed higher ΔF/F signals than the RFA (Fig. 2*C*, left, one-way RM ANOVA with Tukey’s *post hoc* test, *F*_(2,12)_ = 37.35, *p *=* *7.03 × 10^−6^ for the 1P-related hemisphere, *F*_(2,12)_ = 14.1, *p *=* *7.10 × 10^−4^ for the 2P-related hemisphere; Fig. 2*C*, right, one-way RM ANOVA with Tukey’s *post hoc* test, *F*_(2,12)_ = 52.28, *p *=* *1.19 × 10^−6^ for the 1P-related hemisphere, *F*_(2,12)_ = 23.80, *p *=* *6.66 × 10^−5^ for the 2P-related hemisphere, Two-tailed paired *t* test, *p *=* *0.021 for 1P-CFA vs 2P-CFA, *p *=* *0.23 for 1P-RFA vs 2P-RFA, *p *=* *0.062 for 1P-VIS vs 2P-VIS). We also found that two hemispheres represented each corresponding forelimb movements. Compared with the 1P-related hemisphere, the Ca^2+^ signals level of the 2P-related hemisphere was lower in correct and overT incorrect trials but higher in wrongP incorrect trials during the 1P timing. In case of bothP incorrect trials, involved simultaneous movements of both forelimbs, there was no significant difference between the two hemispheres (*p *=* *6.85 × 10^−4^ for correct, *p *=* *2.73 × 10^−5^ for overT, *p *=* *0.367 for bothP, *p *=* *0.0072 for wrongP, two-tailed paired *t* test, Jackknife resampling; Extended Data Fig. 2-1*A*)

10.1523/ENEURO.0200-21.2021.f2-1 Extended Data Figure 2-1 Wide-field Ca^2+^ imaging (WFI) dynamics during the biPress task. ***A***, Comparison of activities between 1P-related hemisphere and 2P-related hemisphere during the 1P. ***B***, Comparison of activities between 1P and 2P in the 1P-related and 2P-related hemisphere (*p *=* *0.573 for 1P-hemisphere, *p *=* *2.80 × 10^−5^ for 2P-hemisphere, two-tailed paired *t* test, Jackknife resampling). ***C***, Activity peak timing in the VIS for beginners (left) and experts (right). In all data, gray lines indicate individual data. All error bars represent SEM; *p <* *0.05, **p <* *0.01, ***p <* *0.001. *Figure Contributions:* Minju Jeong performed the experiments. Hyeonsu Lee analyzed the data. Download Figure 2-1, TIF file.

Interestingly, the sequential peaks of 1P-related and 2P-related cortical activities of both the CFA and RFA was observed in correct trials but not in incorrect trials during the task. This peak interval between 1P-related and 2P-related motor cortical areas was shortened and became less variable at week 3 of training (experts) compared with the those in the beginning of training (beginners), which was consistent with our behavioral patterns [Fig. 2*D–L*; two-tailed paired *t* test, *p *=* *0.41 for 1P-CFA, *p *=* *4.67 × 10^−4^ for 2P-CFA (Fig. 2*D*, left) and two-tailed paired *t* test, *p *=* *0.26 for 1P-RFA, *p *=* *3.35 × 10^−4^ for 2P-RFA (Fig. 2*D*, right); 1P-related hemisphere: 0.0913 s vs 2P-related hemisphere: 1.11 s, *p *=* *0.0156 (Fig. 2*F*); 1P-related hemisphere: 0.134 s vs 2P-related hemisphere: 0.284 s, *p *=* *0.0156 (Fig. 2*H*); 1P-related hemisphere: 0.135 s vs 2P-related hemisphere: 1.21 s, *p *=* *0.0156 (Fig. 2*J*); 1P-related hemisphere: 0.145 s vs 2P-related hemisphere: 0.276 s, *p *=* *0.0313, mice *n* = 7, Wilcoxon signed-rank test (Fig. 2*L*)]. Although the Ca^2+^ signals in the 2P-related hemisphere exhibited arisen during the 1P even before 2P was performed, the signals level was weaker during the 2P than the 1P timing in the correct trials (*p *=* *2.80 × 10^−5^, two-tailed paired *t* test, Jackknife resampling; Extended Data Fig. 2-1*B*). We also confirmed that this sequential activity peak in the correct trials was not observed between left and right hemispheres of the VIS (*n* = 7 mice, Wilcoxon signed-rank test, *p *>* *0.05; Extended Data Fig. 2-1*C*).

### The motor cortex responsible for the 1P also generates motor signals for the 2P by ipsilateral forelimb

The WFI data led us to consider how the activity sequence is organized between the motor cortices. The results suggested that information flow for successful task performance would occur from 1P-motor area (contralateral to the 1P forelimb) to 2P-motor area (contralateral to the 2P forelimb; Fig. 2*E–L*; Extended Data Fig. 2-1*B*). Therefore, we hypothesized that the 1P-motor area might also encode information about the subsequent 2P by the opposite forelimb and transfer these motor signals before the 2P movements are actually generated. In particular, there might exist neurons whose firing rates increase during the period between after 1P and before 2P (1-2P period), and these neural signals would play important roles in continuous 2P performance of the biPress sequence task.

**Figure 3.**
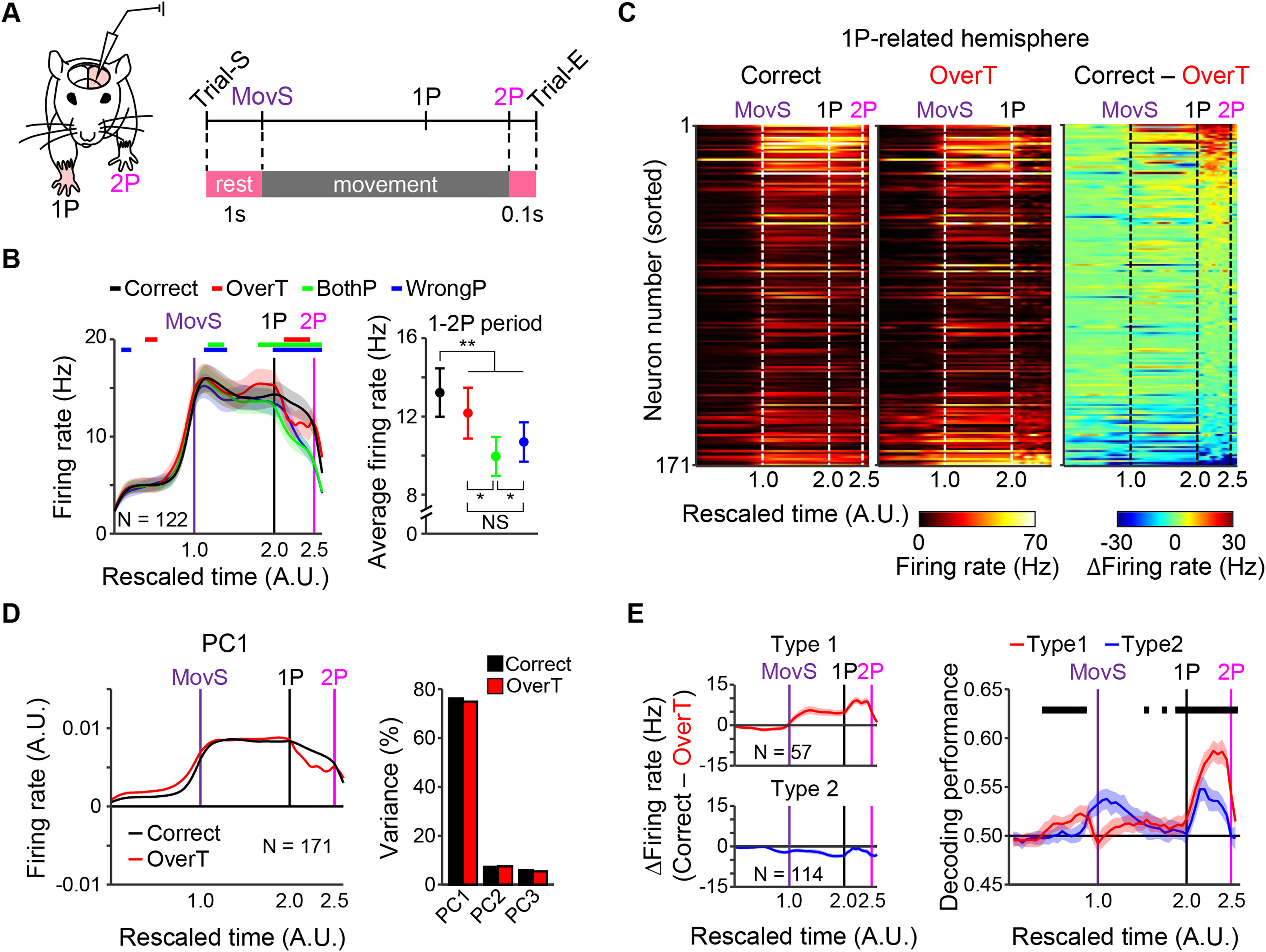
Increased neural activities in the 1P-CFA during the 1-2P period involve in correct performance of the biPress task. ***A***, Schematic illustration of experimental and trial designs. For location of electrodes, see Extended Data Figure 3-1*A*. ***B***, left, Averaged firing rates in correct and incorrect trials. Vertical lines represent the time points of MovS (purple), 1P (black) and 2P (magenta). Colored horizontal bars indicate periods of statistically different firing rate (correct > incorrect). Firing patterns and the ratio of neural population sorted by each behavioral event were shown in Extended Data Figure 3-1*B*,*C*. Right, Averaged firing rates during the interpress interval (for averaged firing rates during the pre-1P periods, see Extended Data Fig. 3-1*D*). Shaded areas and error bars represent SEM. *p < 0.05, **p < 0.01, NS not significant. ***C***, Neural activity profiles sorted by the level of firing rate difference observed during the interpress interval. ***D***, Principal component analysis (PCA) of firing patterns. Left, The first component (PC1). Right, The variance explained by each component. ***E***, Two groups of neurons were classified by their difference in activity between correct and overT incorrect trials. Left, Average activity pattern in each group. Right, Decoding performance analysis. Shaded areas represent SEM. Black horizontal bars indicate statistically different periods (type1 > type2). *Figure Contributions:* Minju Jeong and Youngsoo Kim performed the experiments. Hyeonsu Lee analyzed the data.

To identify the neural signals for correct 2P movements in the sequential biPress task, we performed extracellular single-unit recordings in the 1P-CFA of fully trained mice (Fig. 3*A*; Extended Data Fig. 3-1*A*). Then, we examined and classified firing patterns for each behavioral events as follows: 1 s before the start of movement (MovS; Trial-S), MovS, 1P, 2P, and 0.1 s after 2P (Trial-E; Fig. 3*A*). Since we found that bothP and wrongP incorrect trials were caused by mistaken 1P, we further investigated differences between correct and overT incorrect trials. Most of the 1P-CFA neurons increased their firing rates around the MovS and/or 1P events (82.5%, *n* = 141/171), and there also existed the 1P-CFA neurons which firing rate increased around 2P event (37.4%, *n* = 64/171). Interestingly, these 2P-related neurons showed no such increase during overT trials (Extended Data Fig. 3-1*B*, blue dashed boxes). Therefore, to confirm the strong correlation between these 2P-related activities and correct 2P performance, we scrutinized firing pattern differences between correct and overT incorrect trials.

10.1523/ENEURO.0200-21.2021.f3-1 Extended Data Figure 3-1 *In vivo* extracellular recording during the biPress task. ***A***, Schematic representation of electrode location in the CFA. The locations of recording sites was determined using DiI electrode track and lesion sites (red dots). Anterior-posterior coordinates from bregma were obtained with reference to the Franklin and Paxinos Mouse Brain Atlas. ***B***, Classification of individual units by event-related firing patterns. Colored vertical lines represent the timings of MovS (purple), 1P (black) and 2P (magenta). Note that the 2P-event-responsive neurons showed noticeably different activity patterns between correct and overT incorrect trials (blue dashed boxes). ***C***, The percentage of each classified neuron. ***D***, Averaged firing rates at 1P before 500 ms and after 100 ms in correct and overT/bothP incorrect trials. (Friedman test with Tukey’s *post hoc* test, *p *=* *0.48 for the correct vs overT; *p *=* *0.0016 for the correct vs bothP; *p *=* *0.055 for the overT vs bothP.) Error bars represent SEM; **p <* *0.01. *Figure Contributions:* Minju Jeong performed the experiments. Hyeonsu Lee analyzed the data. Download Figure 3-1, TIF file.

Comparison of activity patterns between correct and overT incorrect trials also showed significant differences during the 1-2P period (Fig. 3*B–D*). BothP and wrongP incorrect trials also showed lower activity than correct trials during the 1-2P period. However, this activity decrease was also observed during the period between MovS and 1P (pre-1P) in bothP and wrongP trials [Wilcoxon signed-rank test, *p *<* *0.05 (Fig. 3*B*, left); Friedman test with Tukey’s *post hoc* test, *p <* *0.05, **p <* *0.01 (Fig. 3*B*, right)]. In addition, to examine whether the neural activity during the 1-2P period could discriminate correct and overT incorrect task performances, we performed a classification test for neural decoding. We firstly classified the 1P-CFA neurons into two groups: those that showed differences between correct and overT incorrect trials (type1) and those that did not (type2; Fig. 3*E*, left). Then, we separately trained a SVM with neural activities of correct and overT incorrect trials of each group, respectively. As expected, this decoding analysis showed higher accuracy in the type1 group than the type2 group during the 1-2P period (two-sample *t* test, *p *<* *0.05; Fig. 3*E*, right), indicating that the decoder could more clearly classify behavioral performance from neurons with the higher activity during the 1-2P periods. Together these results suggest that the 1P-CFA neurons encode neural signals required for a correct performance of 2P during the 1-2P period.

### The 2P-related motor signals from the 1P-CFA neurons are transferred to the 2P-CFA via the corticocortical pathways

**Figure 4.**
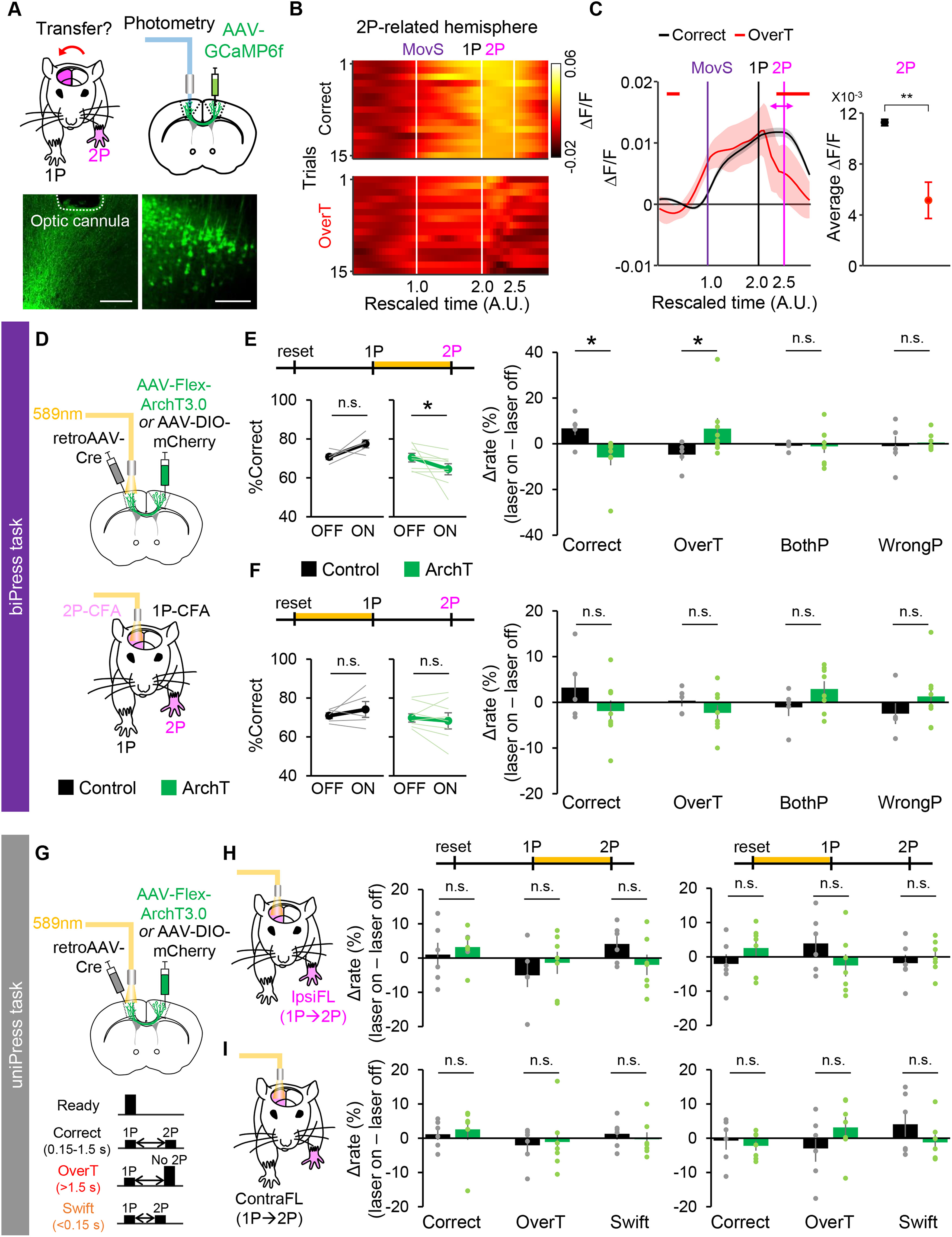
Increased neural activities of corticocortical axon terminals in the 2P-CFA during the 1-2P period involve in correct performance of the biPress task. ***A***, Experimental design of a fiber photometry to image the Ca^2+^ activity of axonal fibers of 1P-CFA neurons in the 2P-CFA. Representative image showing virus-infected CFA neurons and the location of the implanted optic cannula. Scale bars: 500 μm. ***B***, Representative profiles of axonal Ca^2+^ dynamics, presented as a heatmap. ***C***, Average Ca^2+^ dynamics across all mice. Left, Averaged Ca^2+^ activity in correct and overT incorrect trials. Colored horizontal bars indicate the periods with significantly higher activity compared with incorrect trials. Right, Averaged Ca^2+^ firing rates at the 2P timing ([2P – 250 ms, 2P + 250 ms]). Shaded areas and error bars represent 95% confidence interval. ***D***, Experimental scheme for the photoinhibition of corticocortical projections of 1P-CFA neurons during the biPress task. ***E***, Comparisons of the correct rate (left) and differences of rate in correct and each incorrect type trials between optogenetic light off and on (right) by photoinhibition during the interpress interval. ***F***, Comparisons of the correct rate (left) and differences of rate in correct and each incorrect type trials between optogenetic light off and on (right) by photoinhibition during the pre-1P period. For comparisons of latency to 1P, see Extended Data Figure 4-1*A–C*. ***G***, Experimental scheme for the photoinhibition of corticocortical projections of CFA neurons during the timed unimanual-press sequence (uniPress) task by either ipsilateral (ipsiFL; ***H***) or contralateral (contraFL; ***I***) forelimb to the CFA neurons. 2P was performed by the same forelimb with the 1P. Left, Photoinhibition during the 1-2P period. Right, Photoinhibition during the pre-1P period. All data are represented as mean ± SEM, and individual data are represented as light-colored lines or circles; *p <* *0.05, **p <* *0.01, n.s. not significant. For optogenetic inactivation of 1P-CFA neurons, see Extended Data Figure 4-1*D–H*. *Figure Contributions:* Minju Jeong performed all experiments and analyzed the data of optogenetic experiments. Hyeonsu Lee analyzed the data of photometry experiments.

To activate the 2P-CFA neurons of the opposite hemisphere for generation of the 2P forelimb movement, we speculated that the 1P-CFA neurons might send signals through the corticocortical projections during those movement transition periods. To examine this possibility, we measured the axon-terminal Ca^2+^ signals of the 1P-CFA neurons in the 2P-CFA by injecting an AAV-expressing GCaMP6f (AAV-GCaMP6f) into the 1P-CFA and implanting an optic cannula in the 2P-CFA (Fig. 4*A*). Using this system, we observed that the activity of axon terminals of 1P-neurons in the 2P-CFA is highly elevated during 1-2P periods in correct trials (Fig. 4*B*), but not in overT incorrect trials [Mann–Whitney *U* test, *p *<* *0.05 (Fig. 4*C*, left); Mann–Whitney *U* test, *p* = 0.0088 (Fig. 4*C*, right)], suggesting that transfer of motor signals from 1P-CFA to 2P-CFA is important for 2P movements in the biPress task.

10.1523/ENEURO.0200-21.2021.f4-1 Extended Data Figure 4-1 Optogenetic inactivation of the CFA affected biPress performance but did not disturb 1P or 2P forelimb movement. ***A***, Experimental scheme for photoinhibition of cortico-cortical projections of the 1P-CFA neurons during the pre-1P period. ***B***, Differences between optogenetic light on and off of the latency to 1P performed by contralateral (ContraFL, left) or ipsilateral (IpsiFL, right) to CFA neurons. ***C***, Differences between optogenetic light on and off of the number of 1P by ipsiFL. ***D***, Experimental scheme for the photostimulation of inhibitory neurons in the 1P-CFA (contralateral hemisphere of the CFA to the forelimb performing 1P. An optic cannula was implanted into the 1P-CFA area of VGAT-ChR2 mice. ***E***, Comparisons of the correct rate (left) and differences in correct and incorrect rate between optogenetic light off and on (right) by photostimulation during the 1-2P period. ***F***, Comparisons of the correct rate (left; two-tailed paired *t* test, *p *=* *0.793 for control; *p *=* *0.11 for VGAT-ChR2) and differences in correct and incorrect rate between optogenetic light off and on (right; two-tailed unpaired *t* test, *p = *0.159, correct; *p = *0.679 for overT; *p = *0.926 for bothP; *p = *0.111 for wrongP) by photostimulation during the pre-1P period (*n* = 5 littermate control and *n* = 7 VGAT::ChR2 mice). ***G***, Differences between optogenetic light on and off of the latency to 1P performed by contralateral (left; Mann–Whitney *U* test, *p = *0.03, *n* = 5 littermate control and *n* = 7 VGAT-ChR2 mice) or ipsilateral (right; two-tailed unpaired *t* test, *p = *0.644, *n* = 3 littermate control and *n* = 7 VGAT-ChR2 mice) to the CFA implanted an optic cannula. ***H***, Differences between optogenetic light on and off of the number of 1P by ipsiFL (two-tailed unpaired *t* test, *p = *0.745, *n* = 5 litter mate control and *n* = 7 VGAT-ChR2 mice). All data are represented as mean ± SEM, and individual data are represented as light-colored lines or circles; *p <* *0.05. *Figure Contributions:* Minju Jeong performed the experiments and analyzed the data. Download Figure 4-1, TIF file.

To determine functional contribution of neural activity hemispheric transfers during the 1-2P period in the biPress task, we performed the optogenetic inactivation of corticocortical projections. To selectively inhibit the neural activities of the 1P-CFA corticocortical afferents, we injected an AAV harboring genes for the Cre-recombinase-dependent expression of Archaerhodopsin (AAV-Flex-ArchT) into the 1P-CFA, a retrogradely transduced AAV expressing Cre recombinase (retroAAV-Cre) into the 2P-CFA and then implanted an optic cannula into the 2P-CFA (Fig. 4*D*). Photoinhibition of the 1P-CFA axon terminals in the 2P-CFA during the 1-2P period significantly reduced the ratio of correct trials caused by the increase of overT incorrect trials (*n* = 5 mCherry control and *n* = 8 ArchT mice, left, two-tailed paired *t* test, *p *=* *0.082 for mCherry, Wilcoxon signed-rank test, *p *=* *0.016 for ArchT, right, Mann–Whitney *U* test, *p = *0.03 for correct, *p = *0.045 for overT, *p = *0.622 for wrongP, two-tailed unpaired *t* test, *p = *0.947 for bothP; Fig. 4*E*). In contrast, photoinhibition during the pre-1P period did not affect the task performance (*n* = 5 mCherry control and *n* = 8 ArchT mice, left, two-tailed paired *t* test, *p *=* *0.393 for mCherry, *p *=* *0.435 for ArchT, right, two-tailed unpaired *t* test, *p = *0.220 for correct, *p = *0.291 for overT, *p = *0.153 for bothP, *p = *0.305 for wrongP; Fig. 4*F*). This photoinhibition of corticocortical projections also did not interfere with performing 1P by either contralateral or ipsilateral forelimbs in overall [Extended Data Fig. 4-1*A–C*; *n* = 5 mCherry control and *n* = 7 ArchT mice, Mann–Whitney *U* test, *p = *0.26 (Extended Data Fig. 4-1*A*, left) and Mann–Whitney *U* test, *p = *0.432 (Extended Data Fig. 4-1*A*, right); *n* = 5 mCherry control and *n* = 7 ArchT mice, two-tailed unpaired *t* test, *p = *0.864 (Extended Data Fig. 4-1*C*)], suggesting the interruption of correct 2P performance without effect on movement execution by optogenetic inactivation during the 1-2P period.

We observed that the optogenetic inactivation of 1P-CFA neurons during the 1-2P period by photostimulation of Channelrhodopsin 2 (ChR2) expressed in cortical inhibitory interneurons (Zhao et al., 2011; Guo et al., 2014) also significantly reduced the biPress task performance (Extended Data Fig. 4-1*D*,*E*, *n* = 8 littermate control and *n* = 8 VGAT::ChR2 mice, left, Wilcoxon signed-rank test, *p = *0.250 for control, two-tailed paired *t* test, *p = *0.0157 for VGAT-ChR2; right, two-tailed unpaired *t* test, *p = *0.0103 for correct, *p = *0.251 for overT, *p = *0.0109 for bothP, Mann–Whitney *U* test, *p = *0.195 for wrongP). However, it did not increase overT trials but increased bothP incorrect trials. It indicates a specific role of corticocortical projecting neurons in transfer of neural activities inducing 2P after performing 1P. Although further investigation is needed, we assume that this different effect on the type of incorrect trials might be caused because inhibitory interneurons can simultaneously affect throughout the 1P-CFA neurons (Babl et al., 2019) regardless of their encoding information.

To confirm the specific role of corticocortical projections for sequential bimanual movements rather than simply initiation of the next movement sequence, we also tested the optogenetic inactivation effect on sequential unimanual-press task. We expressed the ArchT in one CFA and implanted an optic fiber into the CFA of opposite hemisphere as the previous experiment. Then, we trained mice to sequentially press the same side pedal twice by the single forelimb, which was either contralateral or ipsilateral to the neuronal soma expressing ArchT (Fig. 4*G*). Mice were water-rewarded for the sequential pedal-press between 0.15 and 1.5 s. They could not be rewarded if they pressed the pedal twice within 0.15 s (swift) or only once within 1.5 s (overT). Photoinhibition of corticocortical axon terminals during both 1-2P and pre-1P periods did not change the unimanual-press task performance [Fig. 4H,*I*, *n* = 6 mCherry control and *n* = 7 ArchT mice, two-tailed unpaired *t* test, *p = *0.572 for correct, *p = *0.459 for overT, *p = *0.140 for swift (Fig. 4*H*, left) and two-tailed unpaired *t* test, *p = *0.250 for correct, *p = *0.197 for overT, *p = *0.556 for swift (Fig. 4*H*, right); *n* = 6 mCherry control and *n* = 7 ArchT mice, left, Mann–Whitney *U* test, *p *=* *0.366 for correct, two-tailed unpaired *t* test, *p = *0.837 for overT, *p = *0.541 for swift, right, two-tailed unpaired *t* test, *p = *0.72 for correct, *p = *0.228 for overT, *p = *0.236 for swift (Fig. 4*I*)]. Together, these results further support that the transfer of neural signals via corticocortical projections during movement transition period contributes for coordinating sequential bimanual movements.

## Discussion

The use of left and right limbs with precise sequence and timing is one of the most essential and basic movements to perform various behaviors. Our results show that the neural correlates of sequential bimanual movements can exist within neurons from a single motor cortical hemisphere. In addition, the subsequent transfer of information, presumably to the contralateral cortex through long-range axonal projections (Hagihara et al., 2021), is critical for movement coordination. However, anatomic tracing studies have revealed that many of these efferent neurons contain axons projecting to motor areas such as the striatum and cortex in both hemispheres (Oh et al., 2014; Zingg et al., 2014; Jeong et al., 2016). Therefore, it has been difficult to determine through which cortical pathway information would be transferred to coordinate sequential bimanual movements. Supported by our observation of sequential hemispheric calcium activation patterns from the WFI experiment, we speculate that the motor cortex can coordinate sequential bimanual movements through direct axonal projections to the contralateral motor cortex (Fig. 2). By combining advances in viral tracing and optogenetics, we were able to selectively target the motor cortex-projecting axon terminals of 1P-hemisphere neurons. This approach allows us to specifically examine the corticocortical projections (Fig. 4). Our fiber photometry imaging indicates that information about the 2P movement is present in these axonal terminals (Fig. 4). Importantly, the inactivation of these axon terminals after 1P, but not before, can impair 2P movements. The temporal precision of optogenetics allows us to compare the effects of corticocortical axon terminal inactivation before and after 1P movements, and to rule out the possibility that our experimental manipulations merely interfere with movement execution.

To directly correlate sequential bimanual movements with neural activity, we chose the head-fixation approach to minimize motion artifacts while still allowing mice to move during neuro-imaging or extracellular recording. During the design of the behavioral apparatus, we carefully optimized the pedal position so that the mouse can only reach each pedal with a unilateral forelimb. After each press with enough force, the pedals retracted until the next trial to avoid a double-tap confound. Lastly, to separate the neural correlates of bimanual movements from unilateral movements, we mandated sequential right-and-left presses (or vice versa) by rewarding trials where sequential bimanual presses were performed within a narrow time window.

The rodent neocortex contains several neural pathways to facilitate left and right forelimb movements; conversely, a unilateral movement may reflect the contributions of both cortical hemispheres. Indeed, near simultaneous brain-wide activation is typically observed during movement (Makino et al., 2017; Terada et al., 2018; Mayrhofer et al., 2019). Therefore, we suspected that if sequential bimanual movements were mediated by interhemispheric interactions, then bilateral cortical activation patterns may be different for bimanual versus unilateral movements. In bimanual movements alone, we observed that the hemisphere corresponding to the 1P peaks before the 2P hemisphere (Fig. 2). This order of activation (1P before 2P) raises the possibility that information is propagating from the 1P to the 2P hemisphere; however, we also considered the alternative premise that each cortex operates independently of the other (Li et al., 2016). In particular, we examined two additional possibilities: (1) the *de novo* generation of movement signals independently in each hemisphere (without interactions); and (2) that a central upstream brain region transmits sequential information to the left and right hemispheres.

These alternative possibilities generate testable hypotheses. For example, if movement signals were indeed independently generated within each hemisphere, we would not expect to see any signals corresponding to the 2P movement within the 1P hemisphere. However, our extracellular recording characterization of 1P-hemisphere neurons identifies several classes of neurons that show increased firing rates during 1P movements, 2P movements, or even during both 1P and 2P movements simultaneously (Extended Data Fig. 3-1*B*,*C*). Meanwhile, if a central, upstream controller transmitted information to both 1P and 2P hemispheres, then inactivating neurons in the 1P-hemisphere would not affect the number of 2P movements. However, our results show that 1P-hemisphere inactivation after 1P movement, but not before, significantly reduces the number of 2P movements (Extended Data Fig. 4-1*D–H*). Both of alternative scenarios are on the premise that the 1P and 2P hemispheres independently control each left and right forelimb, and thus, perturbing 1P-hemisphere should have no repercussion on the subsequent 2P forelimb movement.

We also appraised several important caveats of our experiments. For example, the presence of bilateral projecting cortical neurons suggest the possibility that performing 2P by the ipsilateral forelimb to the 1P-CFA would be generated from the contributions of 1P-CFA neurons besides 2P-CFA (Soma et al., 2017). If so then, the neural activity of bothP incorrect trials before 1P are supposed to be the same or higher than overT and correct trials since both contralateral and ipsilateral forelimbs are involved. However, our result showed the opposite: activity of bothP was significantly lower than correct trials (Extended Data Fig. 3-1*D*). Moreover, the neural activities of bothP and wrongP incorrect trials already declined after the onset of movement (MovS), while activity of overT trials sharply dropped only during the 1-2P period (Fig. 3*B*). Therefore, it is difficult to interpret our results may simply be the activity of neurons in the 1P-hemisphere representing ipsilateral forelimb movements for 2P. Consistently, our optogenetic inactivation results also cannot be explained as the inherent interference with ipsilateral movement execution because inactivating neurons in the CFA did not affect ipsilateral forelimb movements performing 1P (Extended Data Fig. 4-1*C*,*H*) and 2P (Fig. 4*H*).

Our data that optogenetic inactivation of corticocortical projections did not alter the performance of unimanual-press sequence task suggest that corticocortical projections may not be involved in simple sequence movements. Since this unimanual sequence task did not require movement interaction between contralateral and ipsilateral forelimbs, it might not require the transfer of motor signals to the opposite cortical hemisphere but rather the transfer to the striatum (Eliassen et al., 1999; Jin et al., 2014; Rothwell et al., 2015; Geddes et al., 2018). Of note, it may be a concern to use the ArchT for axon terminal inhibition because a previous study has observed a paradoxical rebound of the increase of terminal neurotransmitter releases after ArchT activation (Mahn et al., 2016). However, it is caused only after the long-term illumination, but not after short-term illumination protocol (Manita et al., 2015; El-Gaby et al., 2016; Mahn et al., 2016; Ozawa et al., 2017; Uematsu et al., 2017) that we used in this study (1.5 s at most). Thus, we believe that the major effects of our manipulation in this study is reducing, not increasing, the axonal release of neurotransmitter.

In conclusion, our results support a model of direct corticocortical communication to enable the orchestration of sequential bimanual movements. Importantly, while our results highlight the role of corticocortical projections, this does not mean that other axonal projections or other brain regions are unnecessary. It may be valuable to identify downstream regions where information coordinating fine sequential bimanual movements are integrated, processed, and transmitted. To this end, we hope that our newly developed head-fixed paradigm cannot only further support this line of research, but also be broadly applied to other behavioral experiments beyond the motor function involving decision-making and attentional set shifting.

## Synthesis

Reviewing Editor: Mark Laubach, American University Decisions are customarily a result of the Reviewing Editor and the peer reviewers coming together and discussing their recommendations until a consensus is reached. When revisions are invited, a fact-based synthesis statement explaining their decision and outlining what is needed to prepare a revision will be listed below. The following reviewer(s) agreed to reveal their identity: Alex Kwan.

Please address all points that were raised and note that new experiments are not essential but might improve the paper dramatically. The reviewers felt that the paper can progress to publication either way, with or without new experiments. However, if you choose to not do new studies, then please be careful in revising the text to address all interpretational issues that were raised.

For the video analysis that was suggested, you could choose use to DeepLabCut or something like it. (You might find that DeepPoseKit is a faster option.) Alternatively, a simpler analysis was suggested by one of the reviewers: “Perhaps they can show a few (say, 3) trials in which animals make an OverT error when 2P cortex is stimulated (their Fig 3D,E), and also compare that with a few correct trials in the same animal. Also, please show the time frame (relative value compared to e.g., first press, in ms) and in slow motion (e.g, 5x slower). Mark essential events, such as first press, second press, laser on, laser off, etc. A couple of these video clips will be very helpful for readers to get a intuition about what is going on.”

Comments from Reviewer #1

This paper, “Interhemispheric cortico-cortical pathway for sequential bimanual movements in mice", describes a series of experiments to examine the cortical circuit basis of bimanual forepaw movements in mice. There are a number of strengths with this study. The task is very interesting and well-designed. The movie was helpful to visualize the performance. The imaging and optogenetic perturbation experiments were logical. The analyses of these data as well as the behavior were solid. The findings might not be too surprising - that the motor areas in the two hemispheres coordinate to some degree during this bimanual movement task. The optogenetic experiment was key: it showed a mild disruption, so indeed this cortico-cortical pathway contributes, but probably is one among other possible pathways. This is not a knock on the study, as long as the interpretations were grounded accordingly, and I believe this was true in this paper. Overall, I am positive about the study especially regarding the well-grounded experimental design and the analyses.

- This seems like an excellent task to look at motor coordination and motor timing in a head-fixed mouse. Very cool movie.

- The decoding analysis clearly showed that the neural activity in hemisphere associated with 1P carries signals that can predict the subsequent 2P behavior.

- Not sure if the axonal calcium imaging experiment demonstrate convincingly the signal transfer. The calcium signal presumably rises early during motor planning and 1P execution anyways, due to the 1P hemisphere being active for the first press. The calcium signal would be too slow in terms of decay to tell whether the fluorescence between 1-2P is remnant from the first press or truly represent a bridging of that gap. Comparing the Correct versus OverT trials suggest the signals are different also before 1P, not only during the 1-2P gap.

- There are known issues with axonal terminal stimulation using ArchT (Mahn et al., Nat. Neurosci., 2016). The authors should discuss their findings in light of the technical considerations.

- Line 437-438 “Transfer of neural signals via cortico-cortical projections during movement transition period is required for coordinating bimanual movements” - this conclusion seems too strong given that Fig. 3E shows only mild impairment. This results lead one to think that other pathways - such as cortex to ipsilateral versus contralateral striatum, or commissural neurons in the spine cord, could be other routes in which motor signals could pass and coordinate the movements.

- Are the Figure 1-1, 1-2, etc. meant to be supplementary figures? The figures prepared are high-quality and contain interesting data. As eNeuro has no figure limit, many of these figures could be moved to be main figures.

Comments from Reviewer #2

This paper is based on a compelling behavioral paradigm in which head-restrained mice were trained press two pedals sequentially using two forepaws . The behavioral setup seems easy and could be easily adopted by other labs. A head-fixed behavioral system also provides numerous opportunities of optical and electrophysiological recordings, as well as cell-type-specific studies for the future.

One main point that the authors want to make is that there is a flow of activity from one hemisphere to the other during paw switching (e.g., “the motor cortex responsible for the first press also generates motor signals for the second press by ipsilateral forelimb”). Such an activity flow allows (or rather, facilitates) the animal to switch paws and complete sequential pressing successfully. Some perturbation experiment supports this view. In particular, I found the experiment in Fig 3D-E compelling.

Major:

It might be too simple an idea that neural activity first occurs in one hemisphere, which triggers contralateral movement, and then travels to the other hemisphere, triggering contralateral movement corresponding to the second hemisphere. In fact, rarely can one observe sequential propagation of neural activities related to motor and cognition functions. Here, neural activity, measured by Gcamp mesoscopic imaging (Fig 1D), didn’t necessarily show a difference in the rising phase. In fact, activity in the 2P-corresponding area increases non-selectively together with the activity in 1P-region, although the second paw hadn’t press yet. In general, the precise nature of the signal observed in the motor cortex in this paper needs some discussion. Is it related to contralateral movement at all? In particular, two observations need more explanation. First, neural activity increase is observed in both hemispheres with essentially the same dynamics. Second, in spike data, the activity goes up indistinguishably in ‘Correct’ and ‘WrongP’ trials, in which they move opposite paws. I wonder if the authors can discuss more about this.

Terminal inactivation experiment in Fig 3D-E looks good. It would be nice, however, to record the activity of postsynaptic neurons in 2P-corresponding area in vivo during terminal inactivation. In fact, the lack of electrophysiological recordings from 2P-corresponding M1 is unfortunate. It would make the paper much more interesting and thorough.

Another major shortcoming is the lack of video analysis. It would be nice to at least show example of paw movement during optogenetic manipulation.

Minor:

In line 316: “sequential onset” is not an accurate description. The authors were measuring peak (as far as I can tell) instead of onset. These have very different meanings. In fact, since motor cortex receive sensory input, it is to be expected that peak activity in 2P region is delayed compared to that in 1P region. I am actually more surprised that their rising dynamics almost overlap.

In addition, would be nice the write down the time here. Just stating p value is not very informative.

## Author Response

Comments from Reviewer #1

This paper, “Interhemispheric cortico-cortical pathway for sequential bimanual movements in mice”, describes a series of experiments to examine the cortical circuit basis of bimanual forepaw movements in mice. There are a number of strengths with this study. The task is very interesting and well-designed. The movie was helpful to visualize the performance. The imaging and optogenetic perturbation experiments were logical. The analyses of these data as well as the behavior were solid. The findings might not be too surprising - that the motor areas in the two hemispheres coordinate to some degree during this bimanual movement task. The optogenetic experiment was key: it showed a mild disruption, so indeed this cortico-cortical pathway contributes, but probably is one among other possible pathways. This is not a knock on the study, as long as the interpretations were grounded accordingly, and I believe this was true in this paper. Overall, I am positive about the study especially regarding the well-grounded experimental design and the analyses.

We really appreciate for the reviewer’s kind assessments of our data.

1) This seems like an excellent task to look at motor coordination and motor timing in a head-fixed mouse. Very cool movie.

2) The decoding analysis clearly showed that the neural activity in hemisphere associated with 1P carries signals that can predict the subsequent 2P behavior.

Thanks for these positive comments.

3) Not sure if the axonal calcium imaging experiment demonstrate convincingly the signal transfer. The calcium signal presumably rises early during motor planning and 1P execution anyways, due to the 1P hemisphere being active for the first press. The calcium signal would be too slow in terms of decay to tell whether the fluorescence between 1-2P is remnant from the first press or truly represent a bridging of that gap. Comparing the Correct versus OverT trials suggest the signals are different also before 1P, not only during the 1-2P gap.

We agree with the limitation due to the slow decay kinetics of GCaMP6f. However, we believe that the remnant signals owing to the slow decay kinetics during the 1-2P period may not interfere our interpretation. First, we think that both correct and overT incorrect trials involve the 1P therefore, the pattern of signal decaying should be similar between the two if calcium signal of 1-2P period is remnant from the 1P. Second, we re-analyzed using correct trials with more than 180ms of 1-2P periods (inter-press intervals) since it has been reported that the rising time (action potential to peak) and decay time (peak to half peak) of GCaMP6f is 30ms and 150ms, respectively (Chen et al., 2013; Jercog et al., 2016). We observed the same results that activities during 1-2P periods were still higher in correct than overT incorrect trials (R-Fig. 1).

Third, we also found that there is no significant difference in the Ca2+ dynamics between correct and overT trials during neither the period of TrialS-1P nor MovS-1P (pre-1P period) (R-Fig.2A), while Ca2+ signals between correct and wrongP incorrect trials, which may have different motor planning, shows a significant difference during the pre-1P period (R-Fig. 2B).

4) There are known issues with axonal terminal stimulation using ArchT (Mahn et al., Nat. Neurosci., 2016). The authors should discuss their findings in light of the technical considerations.

According to the paper, Mahn et al., Nat. Neurosci., 2016, a significant increase of axon terminals was caused by 5-min-long illumination. They described that sustained proton pump activity induced a pH-dependent calcium influx in axon terminals but if it’s not sustained then, the ArchT could also effectively decrease neurotransmission releases. Consistently, several previous studies (Manita et al., 2015; El-Gaby et al., 2016; Ozawa et al., 2016; Uematsu et al., 2017) using short-term illumination protocols showed successful inhibition of axon terminal activities, which eventually induced behavioral changes. In our current study, the illumination lasted 1.5 sec at most during the 1-2P period therefore, it could presumably inhibit axon erminals of cortico-cortical projections as the paper Manita et al., 2015. We added this point in our discussion.

Line 538-545:

“Of note, it may be a concern to use the ArchT for axon terminal inhibition because a previous study has observed a paradoxical rebound of the increase of terminal neurotransmitter releases after ArchT activation (Mahn et al., 2016). However, it is caused only after the long-term illumination, but not after short-term illumination protocol (Manita et al., 2015; El-Gaby et al., 2016; Mahn et al., 2016; Ozawa et al., 2017; Uematsu et al., 2017) that we used in this study (1.5 sec at most). Thus, we believe that the major effects of our manipulation in this study is reducing, not increasing, the axonal release of neurotransmitter.”

5) Line 437-438 “Transfer of neural signals via cortico-cortical projections during movement transition period is required for coordinating bimanual movements” - this conclusion seems too strong given that Fig. 3E shows only mild impairment. This results lead one to think that other pathways - such as cortex to ipsilateral versus contralateral striatum, or commissural neurons in the spine cord, could be other routes in which motor signals could pass and coordinate the movements.

Thanks for this critical point. We agree with potential roles of other cortical pathways in bimanual movements thus, we already have discussed about it in the discussion section of our manuscript.

Line 547-551:

“Importantly, while our results highlight the role of cortico-cortical projections, this does not mean that other axonal projections or other brain regions are unnecessary. It may be valuable to identify downstream regions where information coordinating fine sequential bimanual movements are integrated, processed, and transmitted.”

We revised the sentence followed by your comment.

Line 452-454:

“Transfer of neural signal via cortico-cortical projections during movement transition period contributes to coordinating bimanual movements.”

6) Are the Figure 1-1, 1-2, etc. meant to be supplementary figures? The figures prepared are high-quality and contain interesting data. As eNeuro has no figure limit, many of these figures could be moved to be main figures.

Thanks for the comment and positive evaluation. We modified figures based on this suggestion.

Comments from Reviewer #2

This paper is based on a compelling behavioral paradigm in which head-restrained mice were trained press two pedals sequentially using two forepaws. The behavioral setup seems easy and could be easily adopted by other labs. A head-fixed behavioral system also provides numerous opportunities of optical and electrophysiological recordings, as well as cell-type-specific studies for the future.

One main point that the authors want to make is that there is a flow of activity from one hemisphere to the other during paw switching (e.g., “the motor cortex responsible for the first press also generates motor signals for the second press by ipsilateral forelimb”). Such an activity flow allows (or rather, facilitates) the animal to switch paws and complete sequential pressing successfully. Some perturbation experiment supports this view. In particular, I found the experiment in Fig 3D-E compelling.

We appreciate the reviewer’s overall assessment and constructive comments on our study.

Major:

1) It might be too simple an idea that neural activity first occurs in one hemisphere, which triggers contralateral movement, and then travels to the other hemisphere, triggering contralateral movement corresponding to the second hemisphere. In fact, rarely can one observe sequential propagation of neural activities related to motor and cognition functions. Here, neural activity, measured by Gcamp mesoscopic imaging (Fig 1D), didn't necessarily show a difference in the rising phase. In fact, activity in the 2P-corresponding area increases non-selectively together with the activity in 1P-region, although the second paw hadn't press yet. In general, the precise nature of the signal observed in the motor cortex in this paper needs some discussion. Is it related to contralateral movement at all? In particular, two observations need more explanation. First, neural activity increase is observed in both hemispheres with essentially the same dynamics. Second, in spike data, the activity goes up indistinguishably in 'Correct' and 'WrongP' trials, in which they move opposite paws. I wonder if the authors can discuss more about this.

In the wide-field imaging experiment, it is difficult to separate calcium signals from between 2P-hemisphere cortical neuron somas and axon terminals of 1P-hemisphere cortical neurons. Since the 2P was not performed in overT incorrect trials, we assume that detected signals might be mainly from axon terminals of 1P-hermisphere cortical neurons. In fact, the amplitude of calcium signals in each hemisphere before normalization showed different levels (R-Fig.3A). We found that the Ca2+ signals level in the 2P-related hemisphere was lower than 1P-related hemisphere during the 1P in correct and overT incorrect trials but not in bothP and wrongP incorrect trials which showed different 1P performances (R-Fig.3B). In addition, the Ca2+ signals in the 2P-related hemisphere showed weaker during the 1P compared to 2P timing in correct trials (R-Fig.3C). Therefore, it seems hard to conclude both hemispheres have the same dynamics in correct trials.

Although activity of 1P-CFA neurons increased even in wrongP trials (R-Fig.4A), which may be able to induce the opposite forelimb movements, the level of activity was significantly lower than correct trials (R-Fig.4B).

Also, we did not distinguish individual body movements for the behavioral event of movement start (MovS). In other words, this MovS event included movements of 1P-related forelimb, which can cause increasing of 1P-CFA neurons but not actually performing 1P because of not enough cortical activities.

We described these points in our revised manuscript.

Line 321-328:

“We also found that two hemispheres represented each corresponding forelimb movements. Compared to the 1P-related hemisphere, the Ca2+ signals level of the 2P-related hemisphere was lower in correct and overT incorrect trials but higher in wrongP incorrect trials during the 1P timing. In case of bothP incorrect trials, involved simultaneous movements of both forelimbs, there was no significant difference between the two hemispheres (Fig. 2-1A, P = 6.85 x 10-4 for Correct, P = 2.73 x 10-5 for OverT, P = 0.367 for BothP, P = 0.0072 for WrongP, Two-tailed paired t-test, Jackknife resampling).”

Line 340-344:

“Although the Ca2+ signals in the 2P-related hemisphere exhibited arisen during the 1P even before 2P was performed, the signals level was weaker during the 2P than the 1P timing in the correct trials (Fig. 2-1B, P = 2.80 x 10-5, Two-tailed paired t-test, Jackknife resampling).”

Line 222-224:

“We did not specifically classify movement types for the MovS in this study. It included either movements of 1P-related or 2P-related forelimb.”

2) Terminal inactivation experiment in Fig 3D-E looks good. It would be nice, however, to record the activity of postsynaptic neurons in 2P-corresponding area in vivo during terminal inactivation. In fact, the lack of electrophysiological recordings from 2P-corresponding M1 is unfortunate. It would make the paper much more interesting and thorough.

Thanks for this suggestion for an interesting experiment. We also agree that the data of in vivo electrophysiology recordings in 2P-CFA neurons would provide us more insights to understand neural mechanisms for sequential bimanual movements. However, because of the technical difficult in performing high level of in vivo recording and optogenetic manipulation simultaneously, we may not be able to perform this experiment in short period of time. Instead, although there are some technical limitations, we believe our wide-field imaging data (Fig. 2) gave a small clue how the two cortical hemispheres are working during the biPress task.

3) Another major shortcoming is the lack of video analysis. It would be nice to at least show example of paw movement during optogenetic manipulation.

Thanks for the comment. We also agree that the video analysis will be helpful for visualizing how optogenetic manipulation would induce incorrect forelimb movements. Unfortunately, however, we did not record a video for these experiments. These experimental procedures are very challenging to perform in short period of time and may not change the major conclusion of this work. Instead, we prepared new representative video with zoomed-in and slow motion in each correct and incorrect trials. The patterns of behavioral phenotype with optogenetic stimulations (Figure 4) are similar as those in video. We also added more detailed behavioral description in the revised manuscript.

We hope our new videos and explanation can provide a better intuition for the sequential bimanual movements.

Line 287-289 :

“The forelimb for 1P was positioned to the pedal and the other forelimb was placed into the floor to be balanced. These positions were switched each other for 2P (Movie 1).”

Line 291-296:

“Although the mice showed similar movement patterns for 1P but did not show any movements after 1P causing overT incorrect trials (Movie 2). Meanwhile, in bothP trials, the mice positioned both forelimbs to each pedal and pressed them almost simultaneously (Movie 3). In wrongP incorrect trials, the 1P-related forelimb was moved but not placed it into the pedal, and then the other forelimb was performed the pedal-press (Movie 4).”

Minor:

In line 316: “sequential onset” is not an accurate description. The authors were measuring peak (as far as I can tell) instead of onset. These have very different meanings. In fact, since motor cortex receive sensory input, it is to be expected that peak activity in 2P region is delayed compared to that in 1P region. I am actually more surprised that their rising dynamics almost overlap.

In addition, would be nice the write down the time here. Just stating p value is not very informative.

Thanks for the comment. We have changed to use “peak” instead of “onset”. We have also described the exact peak timing in the manuscript.

Line 329-343:

“Interestingly, the sequential peaks of 1P- and 2P-related cortical activities of both the CFA and RFA was observed in correct trials but not in incorrect trials during the task. This peak interval between 1P- and 2P-related motor cortical areas was shortened and became less variable at week 3 of training (experts) compared to the those in the beginning of training (beginners), which was consistent with our behavioral patterns (Fig. 2D–L; 2D, Left: Two-tailed paired t-test, P = 0.41 for 1P-CFA, P = 4.67 x 10-4 for 2P-CFA, Right: Two-tailed paired t-test, P = 0.26 for 1P-RFA, P = 3.35 x 10-4 for 2P-RFA; 2F, 1P-related hemisphere: 0.0913 s vs 2P-related hemisphere: 1.11 s, P = 0.0156; 2H, 1P-related hemisphere: 0.134 s vs 2P-related hemisphere: 0.284 s, P = 0.0156; 2J, 1P-related hemisphere: 0.135 s vs 2P-related hemisphere: 1.21 s, P = 0.0156; 2L, 1P-related hemisphere: 0.145 s vs. 2P-related hemisphere: 0.276 s, P = 0.0313, mice n = 7, Wilcoxon signed rank test). Although the Ca2+ signals in the 2P-related hemisphere exhibited arisen during the 1P even before 2P was performed, the signals level was weaker during the 2P than the 1P timing in the correct trials (Fig. 2-1B, P = 2.80 x 10-5, Two-tailed paired t-test, Jackknife resampling).”
