# Supplementary figures and images for: Interhemispheric Cortico-Cortical Pathway for Sequential Bimanual Movements in Mice
Source: eNeuro. 2021 Aug 24;8(4):ENEURO.0200-21.2021. doi: 10.1523/ENEURO.0200-21.2021 (PMC8387156; doi:10.1523/ENEURO.0200-21.2021)

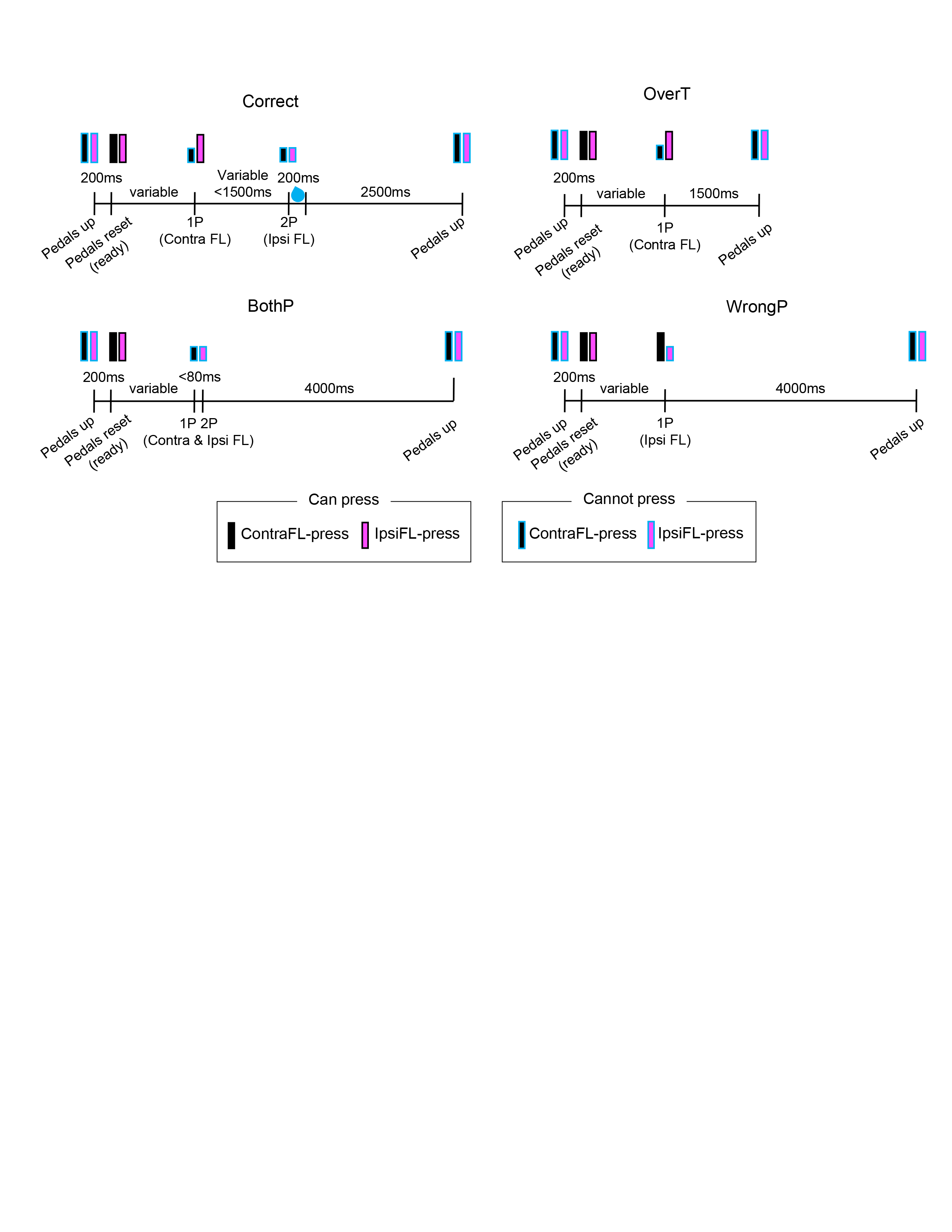

Supplement: Extended Data Figure 1-1 — Diagrams showing pedal movements in the correct and incorrect trials. The pedals are retracted after mice press them until the next trial begins. Contra and Ipsi FL stand for contralateral and ipsilateral forelimb, respectively. Figure Contributions: Minju Jeong performed the experiments. Download Figure 1-1, TIF file. [file enu-eN-NWR-0200-21-s02.tif]

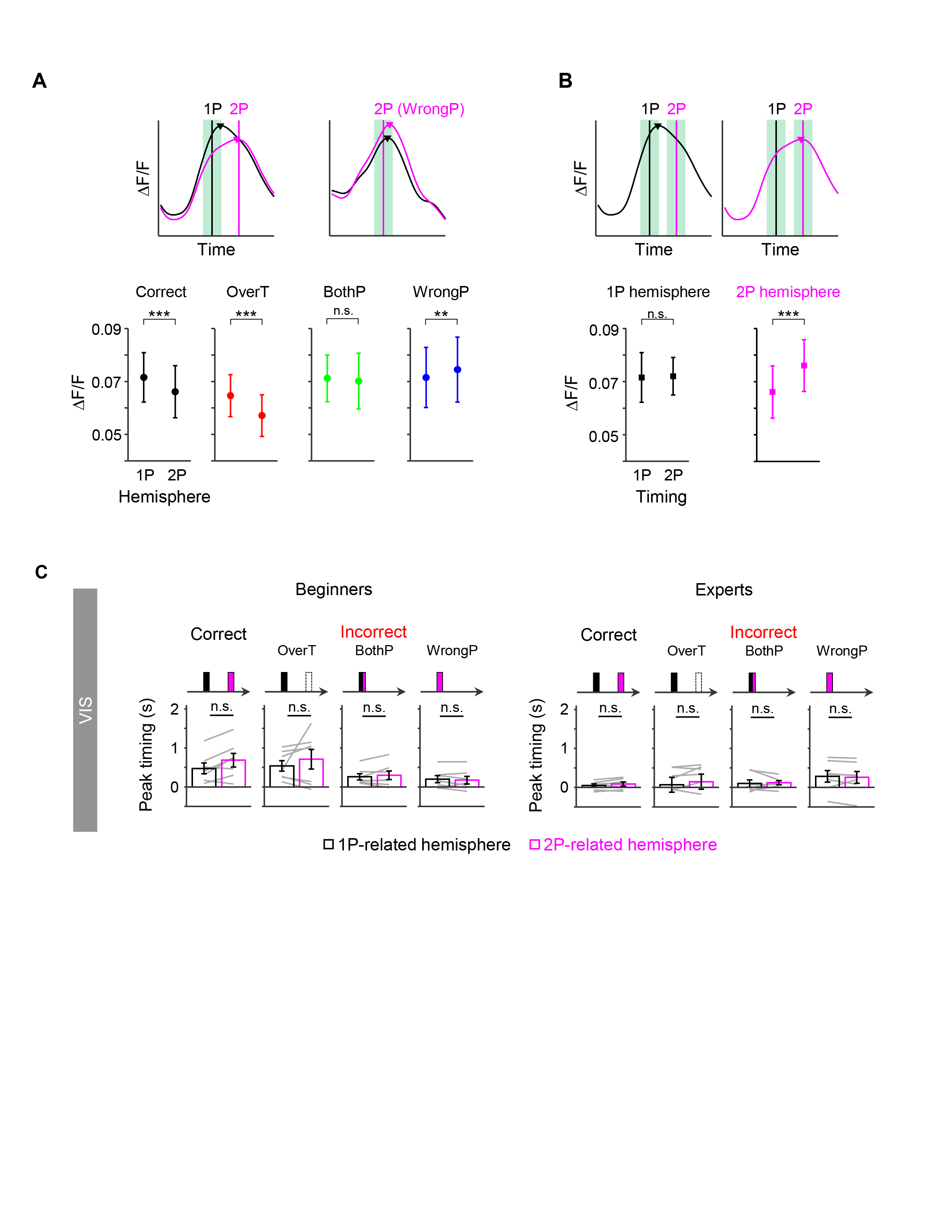

Supplement: Extended Data Figure 2-1 — Wide-field Ca2+ imaging (WFI) dynamics during the biPress task. A, Comparison of activities between 1P-related hemisphere and 2P-related hemisphere during the 1P. B, Comparison of activities between 1P and 2P in the 1P-related and 2P-related hemisphere (p = 0.573 for 1P-hemisphere, p = 2.80 × 10^−5^ for 2P-hemisphere, two-tailed paired t test, Jackknife resampling). C, Activity peak timing in the VIS for beginners (left) and experts (right). In all data, gray lines indicate individual data. All error bars represent SEM; *p < 0.05, **p < 0.01, ***p < 0.001. Figure Contributions: Minju Jeong performed the experiments. Hyeonsu Lee analyzed the data. Download Figure 2-1, TIF file. [file enu-eN-NWR-0200-21-s03.tif]

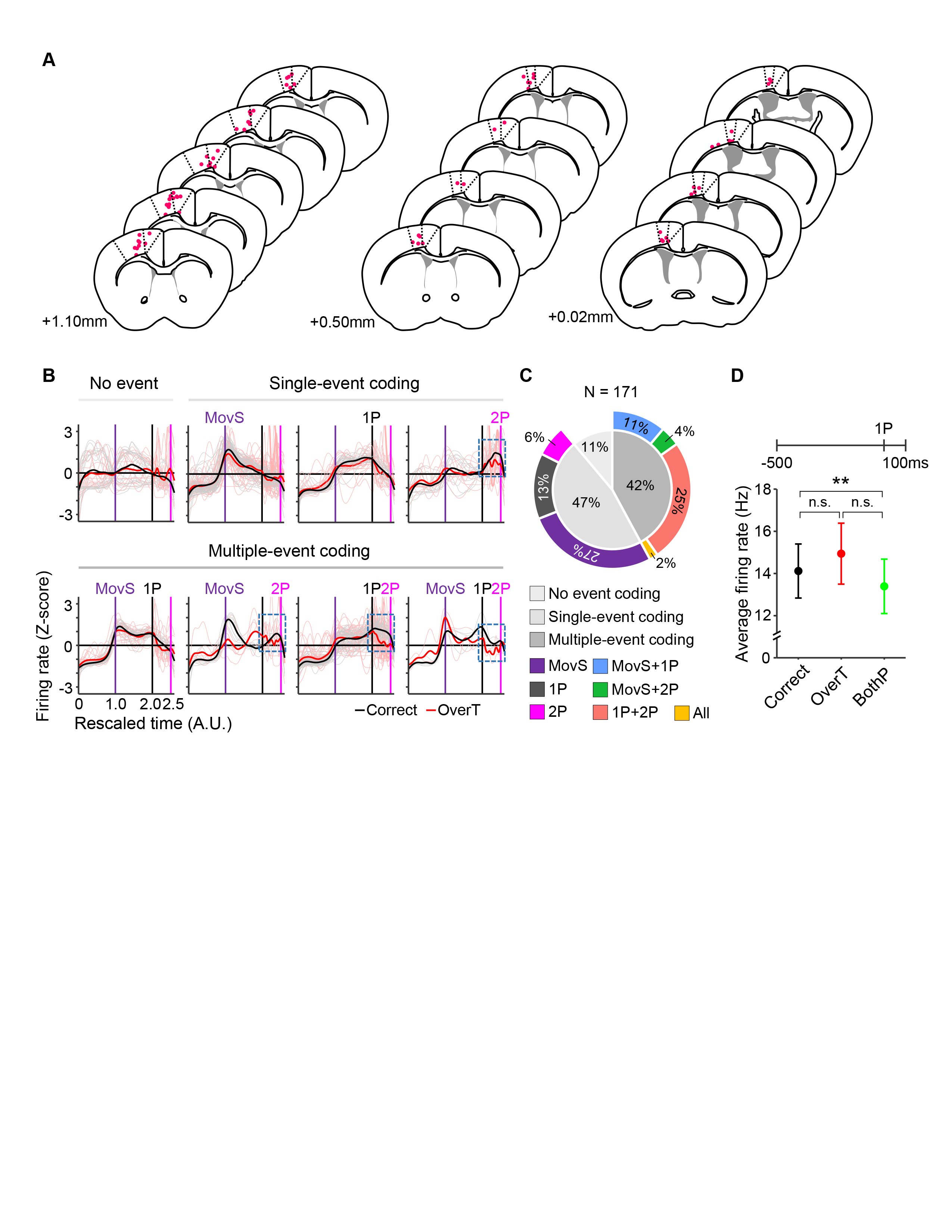

Supplement: Extended Data Figure 3-1 — In vivo extracellular recording during the biPress task. A, Schematic representation of electrode location in the CFA. The locations of recording sites was determined using DiI electrode track and lesion sites (red dots). Anterior-posterior coordinates from bregma were obtained with reference to the Franklin and Paxinos Mouse Brain Atlas. B, Classification of individual units by event-related firing patterns. Colored vertical lines represent the timings of MovS (purple), 1P (black) and 2P (magenta). Note that the 2P-event-responsive neurons showed noticeably different activity patterns between correct and overT incorrect trials (blue dashed boxes). C, The percentage of each classified neuron. D, Averaged firing rates at 1P before 500 ms and after 100 ms in correct and overT/bothP incorrect trials. (Friedman test with Tukey’s post hoc test, p = 0.48 for the correct vs overT; p = 0.0016 for the correct vs bothP; p = 0.055 for the overT vs bothP.) Error bars represent SEM; **p < 0.01. Figure Contributions: Minju Jeong performed the experiments. Hyeonsu Lee analyzed the data. Download Figure 3-1, TIF file. [file enu-eN-NWR-0200-21-s04.tif]

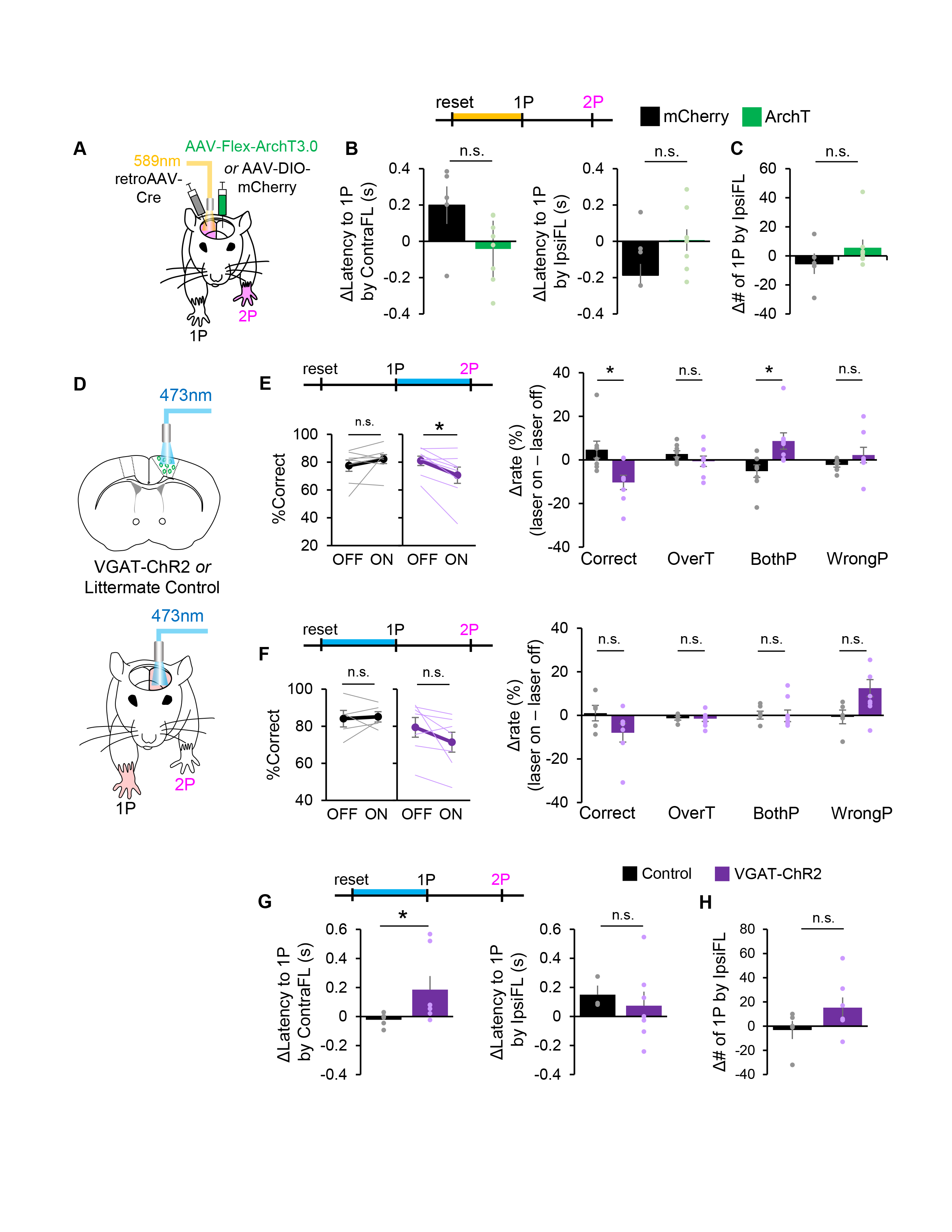

Supplement: Extended Data Figure 4-1 — Optogenetic inactivation of the CFA affected biPress performance but did not disturb 1P or 2P forelimb movement. A, Experimental scheme for photoinhibition of cortico-cortical projections of the 1P-CFA neurons during the pre-1P period. B, Differences between optogenetic light on and off of the latency to 1P performed by contralateral (ContraFL, left) or ipsilateral (IpsiFL, right) to CFA neurons. C, Differences between optogenetic light on and off of the number of 1P by ipsiFL. D, Experimental scheme for the photostimulation of inhibitory neurons in the 1P-CFA (contralateral hemisphere of the CFA to the forelimb performing 1P. An optic cannula was implanted into the 1P-CFA area of VGAT-ChR2 mice. E, Comparisons of the correct rate (left) and differences in correct and incorrect rate between optogenetic light off and on (right) by photostimulation during the 1-2P period. F, Comparisons of the correct rate (left; two-tailed paired t test, p = 0.793 for control; p = 0.11 for VGAT-ChR2) and differences in correct and incorrect rate between optogenetic light off and on (right; two-tailed unpaired t test, p = 0.159, correct; p = 0.679 for overT; p = 0.926 for bothP; p = 0.111 for wrongP) by photostimulation during the pre-1P period (n = 5 littermate control and n = 7 VGAT::ChR2 mice). G, Differences between optogenetic light on and off of the latency to 1P performed by contralateral (left; Mann–Whitney U test, p = 0.03, n = 5 littermate control and n = 7 VGAT-ChR2 mice) or ipsilateral (right; two-tailed unpaired t test, p = 0.644, n = 3 littermate control and n = 7 VGAT-ChR2 mice) to the CFA implanted an optic cannula. H, Differences between optogenetic light on and off of the number of 1P by ipsiFL (two-tailed unpaired t test, p = 0.745, n = 5 litter mate control and n = 7 VGAT-ChR2 mice). All data are represented as mean ± SEM, and individual data are represented as light-colored lines or circles; *p < 0.05. Figure Contributions: Minju Jeong performed the experiments an [file enu-eN-NWR-0200-21-s05.tif]
